# Fault-tolerant control strategy of six-phase permanent magnet synchronous motor based on deadbeat current prediction

**DOI:** 10.1371/journal.pone.0288728

**Published:** 2023-07-19

**Authors:** Hanying Gao, Qi Chen, Shenghan Liang, Yao Dong

**Affiliations:** School of Electrical and Electronic Engineering, Harbin University of Science and Technology, Harbin, China; University of Shanghai for Science and Technology, CHINA

## Abstract

The fault-tolerant control after phase loss is crucial in the studies of the six-phase permanent magnet synchronous motor (PMSM), and the one phase loss is the most frequent phase loss fault. To improve the system instability caused by nonlinear and time-varying perturbations of inductance parameters in a double-Y phase shifted 30° six-phase PMSM, an improved deadbeat predictive current fault-tolerant control (DPC-FTC) method is proposed in this study. The transformation matrix after single-phase open-phase is first reduced and reconstructed, and the reduced-dimensional voltage equation is derived. Based on this equation, the deadbeat current predictive control is then used to predict the expected voltage using the current feedback value and the reference value, so as to shorten the response time and improve the overall control effect. The voltage equation after parameter perturbation is rewritten, and the current discrete transfer function under constant expected voltage before and after parameter perturbation is calculated. Afterwards, to further improve the low stability of fault-tolerant control after phase loss, which is caused by the inductance parameter perturbation of the control system, the weight coefficient is introduced in order to enhance the deadbeat predictive current control so that it splits and optimizes the direct-quadrature axis current. The stability of the system is then analyzed. By changing the weight coefficient, the fault-tolerant control system has a wider stable working range. Finally, the simulation model and experimental platform are completed. The results show that the improved DPC-FTC method improves the permissible inductor parameter uptake range by a factor of 1/β, reduces the current static difference by 32.05% and 46.02% when the inductor parameter is mismatched by a factor of 2, reduces the current oscillation and effectively reduces the sensitivity of system stability to inductor parameter uptake.

## 1 Introduction

In recent years, the fast dynamic response, high power, and high reliability have been the main development directions for the AC motor frequency conversion systems [1]. Compared with the three-phase PMSM, the multiphase motor provides greater freedom, greater redundancy, less torque pulsation, fewer harmonic components in the flux, and less noise [2, 3]. It is widely used in applications requiring high reliability and stability, such as robotics, electric mobility, aerospace engineering, marine propulsion, and wind farms [4–6]. The studies on the improvement of the control effect of the multiphase motor are roughly divided into the optimization of the motor body and the improvement of the control strategy. The efficient control strategies can highlight the advantages of multiphase motors, as previously mentioned. Therefore, the development of high-performance control strategies for multiphase permanent magnet synchronous motors has significant engineering significance. Several studies on control strategies for multiphase motors in normal operation were conducted. They are mainly divided into three categories: vector control, direct torque control, and weak magnetic control. The study presented in [7] focuses on magnetic field-oriented control strategies with vector space decoupling for an asymmetric six-phase PMSM. They simplify the vector space decoupling method to reduce the mathematical operations and increase the operational efficiency. A double Y phase-shifted 30° six-phase permanent magnet synchronous motor is studied in [8]. A direct torque control strategy based on a switching table is used to improve the response of the control system and simplify the control algorithm. The influence of the voltage vector on the stator flux and torque is first analyzed, and the required flux and torque values are calculated according to the motor state variables. The optimum voltage vector is then selected and the motor is finally driven by a PWM module. The method proposed in [9] consists in configuring the parameters of a weak magnetic current PI controller for weak magnetic control of permanent magnet synchronous motors. This method allows the system to perform smooth transitions between weak magnetic and non-weak magnetic regions, and thus ensures a reliable operation. In the control process, there is no need to rely on motor parameters, which can make the system more responsive.

In multi-phase motors, because of the increased number of phases, power devices, and sensors added to the inverter, the potential for system faults significantly increases, while the inverter switching tube faults and single-phase breakage faults have the highest failure rates [10]. Therefore, the fault-tolerant control after motor failure should be considered. The fault-tolerant control strategies for motors following a phase open circuit are broadly divided into two types: the scalar control and the vector space decoupling control. In the former, based on the principle of conservation, the total magnetomotive force remains constant before and after the fault, and the residual phase current reference value is solved under different optimization objectives to perform motor fault-tolerant control. The latter constructs the transformation matrix and develops the mathematical model of the motor after the phase loss to perform the decoupling control. The authors of [11, 12] adjusted the amplitude and phase of the remaining phase currents to account for the motor phase breaking faults. This was performed by considering the principle of constant magneto-dynamic potential before and after the fault, while also imposing different optimization objectives as constraints. However, this approach used hysteresis loop control, which resulted in reducing the dynamic performance of the system. The author of [13] proposes a fault-tolerant control strategy for a dual three-phase motor by deriving the motor model under the dual d-q synchronous rotating coordinate system in the case of an open circuit fault. This strategy performs fault tolerance by controlling the two DC components. The study presented in [14] analyzes the impact of the fifth harmonic space on the fault-tolerant control, and develops mathematical models for the fundamental and fifth harmonic space. It uses the second transformation to implement decoupling control and propose a control strategy which involves injecting fifth harmonic current in order to effectively suppress the torque ripple. However, this approach uses PI control, which leads to a slower dynamic response of the system.

In fault-tolerant control systems, a double closed-loop control strategy is commonly used. This involves using the current loop as the inner loop, which directly affects the performance of the control system. The PI control is the most commonly used current-loop control method. It consists of simple algorithms and has a high robustness [15]. However, its parameters cannot be easily adjusted. On the contrary, the current predictive control develops a mathematical model based on the parameters of the controlled object and different optimization methods, and inputs the phase current and phase voltage parameters into the predictive control algorithm to obtain predicted values. The fast response of its current loop and its reduced motor phase current ripple can greatly enhance the dynamic response of the system. Two main categories of current predictive control exist: the model predictive control and deadbeat control. The authors of [16, 17] proposed to select non-zero vectors according to the system state and combine them with zero vectors to reduce the error between the predicted current and the reference current. In [18], the switching mode problem of PWM inverter is expressed as a model predictive control problem. Considering the switching frequency in steady state, the current transient response of permanent magnet synchronous motor in high-speed domain is achieved. The study presented in [19] proposes a method which performs deadbeat predictive current control by obtaining the expected voltage from the current reference and feedback values, and then using the spatial vector pulse width modulation model to control the inverter, which subsequently regulates the motor. However, discrepancies between predictor variables and expected values can arise due to parameter perturbation. In [20], a finite control set MPC based on virtual vectors is used to predict current control and is applied to the phase deficiency fault of the six-phase motor. By analyzing the voltage offset after the missing phase and compensating the voltage vector, the accuracy of voltage vector selection is improved. Meanwhile, the harmonic current in the x-y subspace is reduced by synthesizing the virtual vector. However, MPC based on fault tolerant control schemes is heavily dependent on the parameters of the multiphase motor and is poorly adapted to the range of parameter variations.

The predictive current control algorithm relies on model derivation, and therefore it is sensitive to the model parameters. During the motor operation, the actual parameters can change due to thermal and saturation effects, which leads to a shift in the system operating performance in practical applications [21]. In order to improve the robustness of the system and ensure a high control performance, several predictive control improvement methods were proposed. They are roughly divided into two strategies: limiting parameter changes and identifying and compensating based on disturbance parameters. The authors of [22] proposed to improve the prediction model by considering the effect of the motor parameter variations, adding a limiting factor, taking the average of the parameter variation coefficients, and predicting the current for each sampling cycle. The authors of [23] considered the sampled current as the predicted current value at the previous moment, and used the recursive least squares method to identify the inductor in order to ensure a fast system response, while considering only the inductor parameter perturbations. In [24], a hyperbolic tangent function is proposed to replace the gain of Luenberger disturbance observer, which can effectively suppress the influence of the peak overcompensation. However, the parameters of the disturbance observer are difficult to adjust and the control system is complex. In [25], a combination of stator current and perturbation observer with predictive control is proposed to use stator prediction instead of sampling current. This allows to compensate for the delay and to use the estimated parameter perturbation in order to compensate for the predicted voltage feedforward. However, the observer design is more complex. In [26], reviewed the robust operation of MPC, including improved prediction models, error estimation compensation schemes and parameter identification schemes, integrated weighting factors, cost functions and adaptive compensation of stator current prediction errors to suppress the parameter mismatch problem, all of the above methods achieve the purpose of improving the stability performance of the control system, but the combined control of parameter identification and compensation also leads to system complexity and However, the combination of parameter identification and compensation control also leads to an increase in system complexity and computation, and does not take into account the problem of incomplete coupling of fault-tolerant control voltages. Therefore, in fault-tolerant control research, improving the stability of fault-tolerant control systems under parameter ingestion and reducing the computational complexity and improving the real-time performance of the system are the keys to current predictive control research. Considering that inductor parameters have the greatest influence on system stability, this study addresses the problem of inductor parameter ingestion.

In this paper, an improved deadbeat current predictive fault-tolerant control algorithm for six-phase PMSM with double-Y phase shift of 30° is proposed in the case of motor single-phase fault. The mathematical model of a double-Y phase shift 30° six-phase PMSM in normal operation is first derived. The dimensional reduction mathematical model of the motor is then reconstructed based on its spatial distribution for each phase and the spatial decoupling after single-phase phase loss. The dimensional reduction voltage equation is finally determined. In order to perform complete decoupling control, the two sides of the equation are multiplied by *M*(*θ*) for secondary transformation. Afterwards, in order to increase the proportion of the control algorithm in the system control cycle and reduce the delay, a deadbeat current predictive control algorithm is used to discretize the dimensional reduction voltage equation after the second transformation. This involves predicting the expected voltage based on the motor current reference value and current feedback value. The equation after the perturbation of inductance parameters is rewritten while considering the nonlinear and time-varying perturbation of the motor inductance parameters during motor fault operation. The current discrete transfer function under the condition of equal expected voltage before and after the perturbation of the inductance parameters is then calculated, and its stability is analyzed. Considering the influence of the parameter perturbation on the closed-loop system, weighting coefficients are added to split and optimize the direct-quadrature axis currents at the current moment and substitute them into the deadbeat current prediction algorithm. Consequently, the discrete transfer function pole distribution of the current under the condition of constant desired voltage before and after parameter perturbation is related to the weight coefficients, and the range of inductor parameter perturbation in the case of stable fault-tolerant control system is increased by changing the size of the weight coefficients. Finally, a simulation experiments is established to evaluate the feasibility of the proposed improved deadbeat predictive current fault-tolerant control method, which effectively reduces the sensitivity of system stability to parameter perturbation.

## 2 Mathematical model of the six-phase PMSM

### 2.1 Mathematical model in normal operation

**Fig 1:**
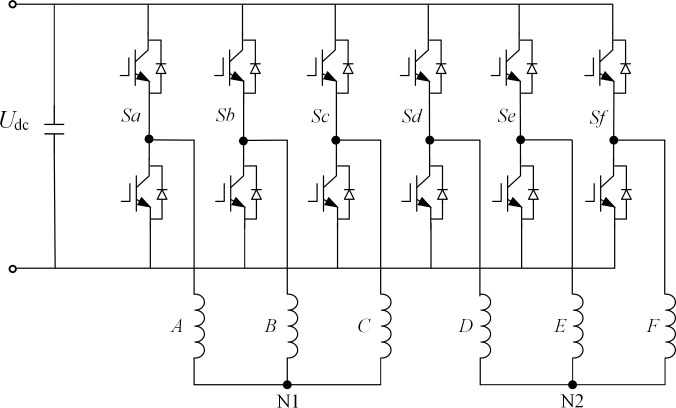
Topological structure of asymmetric six-phase PMSM drive system.

**Fig 2:**
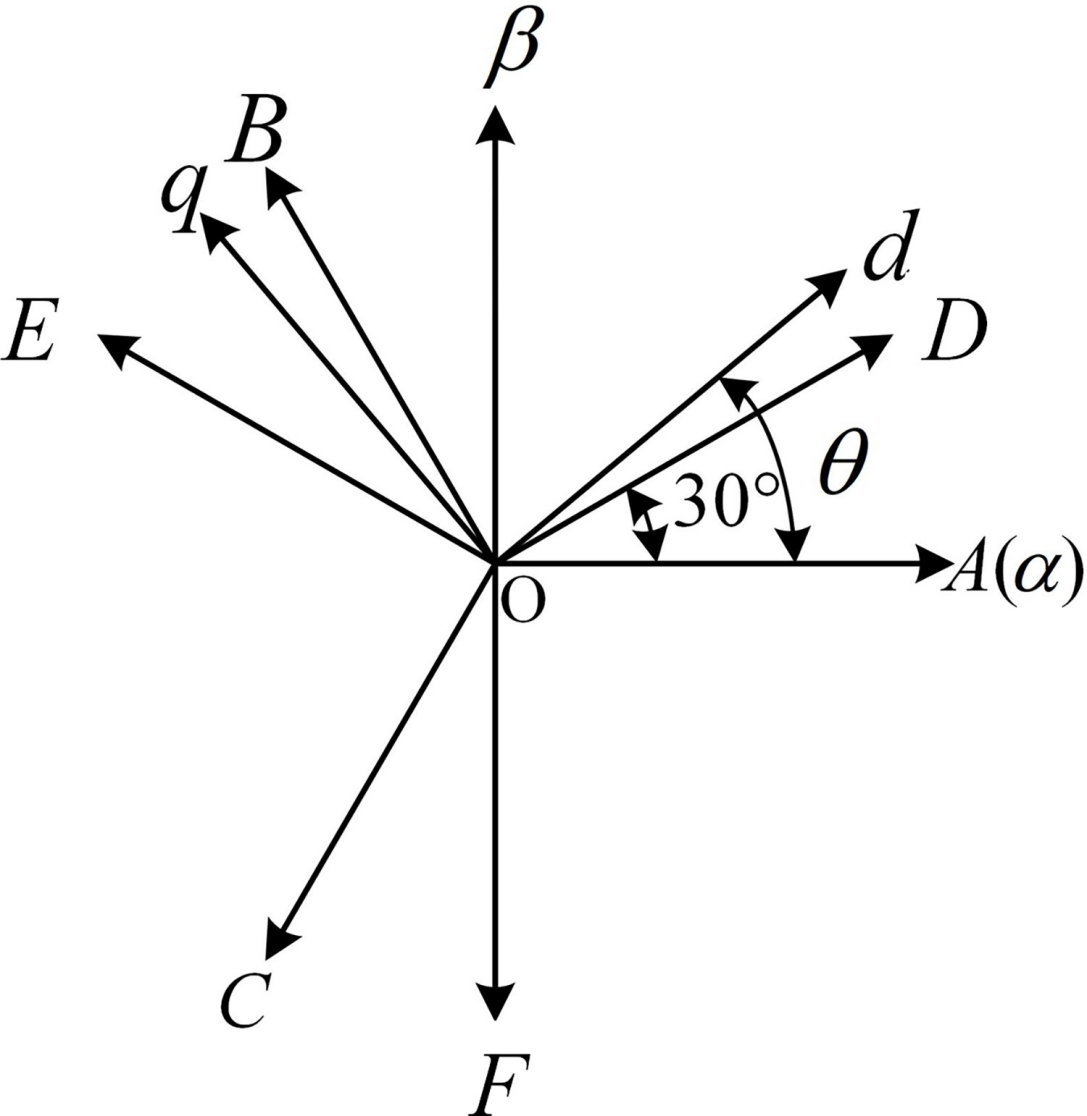
Asymmetric six-phase PMSM winding space structure diagram.

Fig 1 shows the topology structure of the asymmetrically distributed six-phase PMSM drive system, which can be regarded as consisting of two sets of stator windings with a phase difference of 30° between each phase, also known as "double-Y phase shift 30° six-phase PMSM". Fig 2 shows the spatial structures of the two sets of windings, ABC and DEF.

The voltage and magnetic chain equations for a double-Y phase shift 30° six-phase PMSM in the natural coordinate system are given by:

Stator armature winding voltage equation:

$$u_{6s}=R_{6s}i_{6s}+\frac{d}{dt}\psi_{6s}$$ (1)

Stator armature winding flux equation:

$$\psi_{6s}=L_{6s}i_{6s}+\psi_{f}F_{6s}(\theta )$$ (2)

Where *u*_6*s*_ is the phase voltage matrix expressed as $u_{6s}={\left[\begin{array}{cccccc} u_{A} & u_{B} & u_{C} & u_{D} & u_{E} & u_{F} \end{array}\right]}^{T}$, *i*_6*s*_ is the phase current matrix expressed as $i_{6s}={\left[\begin{array}{cccccc} i_{A} & i_{B} & i_{C} & i_{D} & i_{E} & i_{F} \end{array}\right]}^{T}$, *ψ*_6*s*_ is the magnetic chain matrix expressed as $\psi_{6s}={\left[\begin{array}{cccccc} \psi_{A} & \psi_{B} & \psi_{C} & \psi_{D} & \psi_{E} & \psi_{F} \end{array}\right]}^{T}$, *L*_6*s*_ is the stator inductance coefficient matrix, *R*_6*s*_ is the stator resistance matrix expressed as $R_{6s}=R*I_{6}$, *F*_6*s*_ is the phase voltage matrix expressed as $F_{6s}={\left[\begin{array}{cccccc} \cos(\theta ) & \cos(\theta -\frac{2}{3}\pi ) & \cos(\theta -\frac{4}{3}\pi ) & \cos(\theta -\frac{1}{6}\pi ) & \cos(\theta -\frac{5}{6}\pi ) & \cos(\theta -\frac{3}{2}\pi ) \end{array}\right]}^{T}$, and *I*_6_ is a unit matrix of order six.

Due to the high-order strong coupling of the multiphase motor, the variables of the PMSM are mapped to the α-β sub-plane and the generalized zero-sequence sub-plane based on vector space decoupling. For the double-Y phase shift 30° six-phase PMSM the multiphase stationary decoupling theory cannot be directly used, and thus the Clark transform theory is first used to obtain the stationary transformation matrix:

$$C_{6s}=\frac{1}{3}[\begin{array}{cccccc} 1 & -\frac{1}{2} & -\frac{1}{2} & \frac{\sqrt{3}}{2} & -\frac{\sqrt{3}}{2} & 0\\ 0 & \frac{\sqrt{3}}{2} & -\frac{\sqrt{3}}{2} & \frac{1}{2} & \frac{1}{2} & -1\\ 1 & -\frac{1}{2} & -\frac{1}{2} & -\frac{\sqrt{3}}{2} & \frac{\sqrt{3}}{2} & 0\\ 0 & -\frac{\sqrt{3}}{2} & \frac{\sqrt{3}}{2} & \frac{1}{2} & \frac{1}{2} & -1\\ 1 & 1 & 1 & 0 & 0 & 0\\ 0 & 0 & 0 & 1 & 1 & 1 \end{array}]$$ (3)

Using the transformation matrix of Eq (3), the variables of the double-Y phase shift 30° six-phase PMSM can be mapped onto three mutually orthogonal sub-space planes: α-β, z_1_-z_2_, and o_1_-o_2_. More precisely, the first two rows of the matrix correspond to the α-β subspace, the middle two rows correspond to the z_1_-z_2_ subspace, and the last two rows correspond to the o_1_-o_2_ zero-order subspace.

In addition, there are two sets of winding neutral point isolation, the o_1_-o_2_ subspace corresponding variable component is null, the winding magnetic dynamic potential before and after the α-β stationary coordinate transformation is unchanged, and only the current components corresponding to the α-β subspace participate in the electromechanical energy conversion. Therefore, only the α-β subspace is transformed by rotating coordinate transformation, and the rest of the subspace is used to maintain the stationary transformation. The transformation matrix is given by:

$$P_{6s}=[\begin{array}{ccc} \cos\theta & \sin\theta & 0\\ -\sin\theta & \cos\theta & 0\\ 0 & 0 & I_{4} \end{array}]$$ (4)

Thus, the total transformation matrix is *T* = *P*_6s_*C_6s_.

### 2.2 Mathematical model of the one-phase open circuit

When the motor runs open circuit in one phase, if the static transformation matrix is not changed, the current is no longer decoupled in the fundamental and harmonic subplanes, which causes torque pulsations. The transformation matrix after the one phase open circuit is reconstructed in accordance with the vector space decoupling theory, and the relevant variables of the remaining phase are transformed into the α-β fundamental wave subspace in electromechanical energy conversion and the mutually orthogonal z_1_-z_2_-z_3_ subspace. Considering the F-phase open circuit as an example, as one phase of the motor is open circuit, the transformation matrix is downgraded to a fifth-order matrix:

$$C_{5s}=\frac{1}{3}{\left[\begin{array}{ccccc} \alpha & \beta & z_{1} & z_{2} & z_{3} \end{array}\right]}^{T}$$ (5)

According to the principle of constant magnetomotive force generated by the winding and the spatial distribution of the winding, the α and β matrices can be expressed as follows:

$$\begin{array}{c} \alpha ={\left[1\;-\frac{1}{2}\;-\frac{1}{2}\;\frac{\sqrt{3}}{2}\;-\frac{\sqrt{3}}{2}\right]}^{T}\\ \beta ={\left[0\;\frac{\sqrt{3}}{2}\;-\frac{\sqrt{3}}{2}\;\frac{1}{2}\;\frac{1}{2}\right]}^{T} \end{array}$$ (6)

After the missing phase β and z_1_ are no longer orthogonal, the principle of mutual orthogonality of the subspaces is considered, the β matrix is simplified and combined with the current constraint after the missing phase of one phase. The reduced order stationary transformation matrix after the missing phase can then be obtained:

$$C_{5s}=[\begin{array}{ccccc} 1 & -\frac{1}{2} & -\frac{1}{2} & \frac{\sqrt{3}}{2} & -\frac{\sqrt{3}}{2}\\ 0 & \frac{\sqrt{3}}{2} & -\frac{\sqrt{3}}{2} & 0 & 0\\ 1 & -\frac{1}{2} & -\frac{1}{2} & -\frac{\sqrt{3}}{2} & \frac{\sqrt{3}}{2}\\ 1 & 1 & 1 & 0 & 0\\ 0 & 0 & 0 & 1 & 1 \end{array}]$$ (7)

The rotation coordinate matrix is expressed as:

$$P_{5s}=[\begin{array}{ccc} \cos\theta & \sin\theta & 0\\ -\sin\theta & \cos\theta & 0\\ 0 & 0 & I_{3} \end{array}]$$ (8)

Similar to the modelling analysis during the normal operation of the motor, the variables associated with the stator winding voltage equation and the magnetic chain equation when one phase is missing are removed from the rows or columns of the corresponding phase according to their spatial distribution in the winding. The remaining phase variables are similar to those during normal operation. The transformation matrix is substituted into the two ends of the voltage and magnetic chain equations after phase loss:

$$\begin{array}{l} T\Psi_{s}=TL_{5s}I_{5s}+T\varphi_{f}F_{5s}(\theta )\\ \;=(TL_{5s}T^{-1})•TI_{5s}+\varphi_{f}•TF_{5s}(\theta )\\ \;=L_{dqs}I_{dq}+\varphi_{f}F_{dq} \end{array}$$ (9)

$$\begin{array}{l} TU_{5s}=TR_{5s}I_{5s}+T\frac{d\Psi_{s}}{dt}\\ \;=(TR_{5s}T^{-1})•TI_{5s}+\frac{d[T\Psi_{s}]}{dt}-\frac{dT}{dt}\Psi_{s}\\ \;=(TR_{5s}T^{-1})•TI_{5s}+\frac{d[T\Psi_{s}]}{dt}-\frac{dT}{dt}T^{-1}•T\Psi_{s}\\ \;=R_{dq}I_{dq}+\frac{dL_{dqs}}{dt}I_{dq}+L_{dqs}\frac{dI_{dq}}{dt}+\Psi_{f}\frac{dF_{dq}}{dt}-\Omega (L_{dqs}I_{dq}+\Psi_{f}F_{dq})\\ \;=R_{dq}I_{dq}+L_{dqs}\frac{dI_{dq}}{dt}+(\frac{dL_{dqs}}{dt}I_{dq}-\Omega L_{dqs})I_{dq}+\Psi_{f}(\frac{dF_{dq}}{dt}-\Omega F_{dq}) \end{array}$$ (10)

After the motor is out of phase, the last row and last column of the inductance coefficient matrix are removed, and the rest is similar to the normal operation:

$$L_{5s}=L_{m}[\begin{array}{ccccc} 1 & -\frac{1}{2} & -\frac{1}{2} & \frac{\sqrt{3}}{2} & -\frac{\sqrt{3}}{2}\\ -\frac{1}{2} & 1 & -\frac{1}{2} & 0 & \frac{\sqrt{3}}{2}\\ 1 & -\frac{1}{2} & 1 & -\frac{\sqrt{3}}{2} & 0\\ \frac{\sqrt{3}}{2} & 0 & -\frac{\sqrt{3}}{2} & 1 & -\frac{1}{2}\\ -\frac{\sqrt{3}}{2} & \frac{\sqrt{3}}{2} & 0 & -\frac{1}{2} & 1 \end{array}]+L_{z}I_{5}$$ (11)

where *I*_5_ is a unit matrix of order 5.

The velocity matrix is given by:

$$\Omega =\omega [\begin{array}{cc} 0 & 1\\ -1 & 0 \end{array}]$$ (12)

The *u*_*d*_/*u*_*q*_ equation after dimension reduction is obtained by substituting Eqs (7)and (8) into Eqs (9) and (10). Considering that only the components of the α-β fundamental subspace participate in the electromechanical energy conversion, only the components in the d-q coordinate system are retained. In order to further simplify the equation calculation, the variables are simplified to two-dimensional variables. That is, the reduced dimensional voltage equation after the F-phase open circuit is given by:

$$\begin{array}{l} \left[\begin{array}{c} u_{d}\\ u_{q} \end{array}\right]=\left[\begin{array}{cc} R & 0\\ 0 & R \end{array}\right]\left[\begin{array}{c} i_{d}\\ i_{q} \end{array}\right]+\left(M(\theta )\left[\begin{array}{cc} 3L_{d} & 0\\ 0 & 3L_{q} \end{array}\right]+\left[\begin{array}{cc} L_{z} & 0\\ 0 & L_{z} \end{array}\right]\right)\frac{d}{dt}\left[\begin{array}{c} i_{d}\\ i_{q} \end{array}\right]\\ \;-\omega \left(M(\theta )\left[\begin{array}{cc} 0 & 3L_{q}\\ -3L_{d} & 0 \end{array}\right]+\left[\begin{array}{cc} 0 & L_{z}\\ -L_{z} & 0 \end{array}\right]\right)\left[\begin{array}{c} i_{d}\\ i_{q} \end{array}\right]+\omega N(\theta )\varphi_{f} \end{array}$$ (13)

where *L*_*d*_ and *L*_*q*_ respectively represent the equivalent inductance in the d and q axes, *L*_*z*_ respectively represents the leakage inductance of the motor, *R*, *ω*, and *φ*_*f*_ respectively denote the magnitude of the motor stator resistance, the electromechanical angular velocity, and the value of the motor’s permanent magnet chain, *M*(*θ*) and *N*(*θ*) are given by:

$$\{\begin{array}{c} M(\theta )=[\begin{array}{cc} 0.75+0.25\cos(2\theta ) & -0.25\sin(2\theta )\\ -0.25\sin(2\theta ) & 0.75-0.25\cos(2\theta ) \end{array}]\\ N(\theta )=[\begin{array}{c} -0.25\sin(2\theta )\\ 0.75-0.25\cos(2\theta ) \end{array}] \end{array}$$ (14)

The voltage equation shown in Eq (13) has the same effect as the voltage equation of the motor in normal operation. Therefore, Eq (13) can be rewritten as a common voltage equation for the motor.

The dimensionality reduction fault-tolerant algorithm is used in this paper. If the motor has other phase open circuit faults, the analysis method is similar to the F-phase open circuit. The remaining five-dimensional vectors in the transformation matrix *C*_5*s*_ need to be first corrected, where α and β fundamental vectors can be obtained from the transformation matrix *C*_5*s*_, z_1_ and z_2_ harmonic plane vectors can be calculated from α and β fundamental vectors, z_3_ zero sequence plane vectors are derived by combining the current constraints after one phase open circuit. In addition, the inductance coefficient matrix should be corrected by removing the corresponding rows and columns of the inductance coefficient matrix after phase loss according to the spatial distribution of the motor inductance. Finally, the transformation matrix and the inductance coefficient matrix are substituted into the voltage and magnetic chain equations after one phase open circuit, and the voltage component equations in the d-q coordinate system are obtained.

### 2.3 Mathematical model of the one-phase open circuit based on quadratic transformation

It can be seen from Eq (13) that the mathematical model of the double-Y phase shift 30° six-phase PMSM, with a coefficient matrix containing variables that change depending on the time, is not fully decoupled. Therefore, the effect of the time should be eliminated. Thus, to achieve a complete decoupling of the motor, a quadratic transformation is performed by simultaneously multiplying the two sides of the voltage equation by *M*(*θ*):

$$\begin{array}{l} M^{-1}(\theta )\left[\begin{array}{c} u_{d}\\ u_{q} \end{array}\right]=M^{-1}(\theta )\left[\begin{array}{cc} R & 0\\ 0 & R \end{array}\right]\left[\begin{array}{c} i_{d}\\ i_{q} \end{array}\right]+\left(\left[\begin{array}{cc} 3L_{d} & 0\\ 0 & 3L_{q} \end{array}\right]+M^{-1}(\theta )\left[\begin{array}{cc} L_{z} & 0\\ 0 & L_{z} \end{array}\right]\right)\frac{d}{dt}\left[\begin{array}{c} i_{d}\\ i_{q} \end{array}\right]\\ \;-\omega \left(\left[\begin{array}{cc} 0 & 3L_{q}\\ -3L_{d} & 0 \end{array}\right]+M^{-1}(\theta )\left[\begin{array}{cc} 0 & L_{z}\\ -L_{z} & 0 \end{array}\right]\right)\left[\begin{array}{c} i_{d}\\ i_{q} \end{array}\right]+M^{-1}(\theta )\omega N(\theta )\psi_{f} \end{array}$$ (15)

By substituting *M* ^-1^ (*θ*) into Eq (15) and simplifying, the following is obtained:

$$\begin{array}{l} \left[\begin{array}{c} u_{u}\\ u_{v} \end{array}\right]=\left(\left[\begin{array}{cc} 1.5R & 0\\ 0 & 1.5R \end{array}\right]+0.5RA(\theta )\right)\left[\begin{array}{c} i_{d}\\ i_{q} \end{array}\right]+\left(\left[\begin{array}{cc} 1.5L_{z}+3L_{d} & 0\\ 0 & 1.5L_{z}+3L_{q} \end{array}\right]+0.5L_{z}A(\theta )\right)\frac{d}{dt}\left[\begin{array}{c} i_{d}\\ i_{q} \end{array}\right]\\ \;+\omega \left(\left[\begin{array}{cc} 0 & -1.5L_{z}-3L_{q}\\ 1.5L_{z}+3L_{d} & 0 \end{array}\right]+0.5L_{z}B(\theta )\right)\left[\begin{array}{c} i_{d}\\ i_{q} \end{array}\right]+\omega \psi_{f}\left[\begin{array}{c} 0\\ 1 \end{array}\right] \end{array}$$ (16)

Where: $A(\theta )=[\begin{array}{cc} -\cos(2\theta ) & \sin(2\theta )\\ \sin(2\theta ) & \cos(2\theta ) \end{array}],\;B(\theta )=[\begin{array}{cc} \sin(2\theta ) & \cos(2\theta )\\ \cos(2\theta ) & -\sin(2\theta ) \end{array}]$.

In Eq (16), the intermediate variables *u*_*u*_ and *u*_*v*_ allow the d and q axis voltage equations to contain no variables that are unrelated to themselves, which reduces the coupling effect between the d and q axis reduced-dimensional voltage equations of the motor. When the other phases of the motor are open-circuited, similar to the F-phase open circuit derivation, a second transformation is required to eliminate the effect of time.

## 3 Fault-tolerant control based on deadbeat current prediction

### 3.1 Fault-tolerant control of traditional deadbeat current prediction

**Fig 3:**
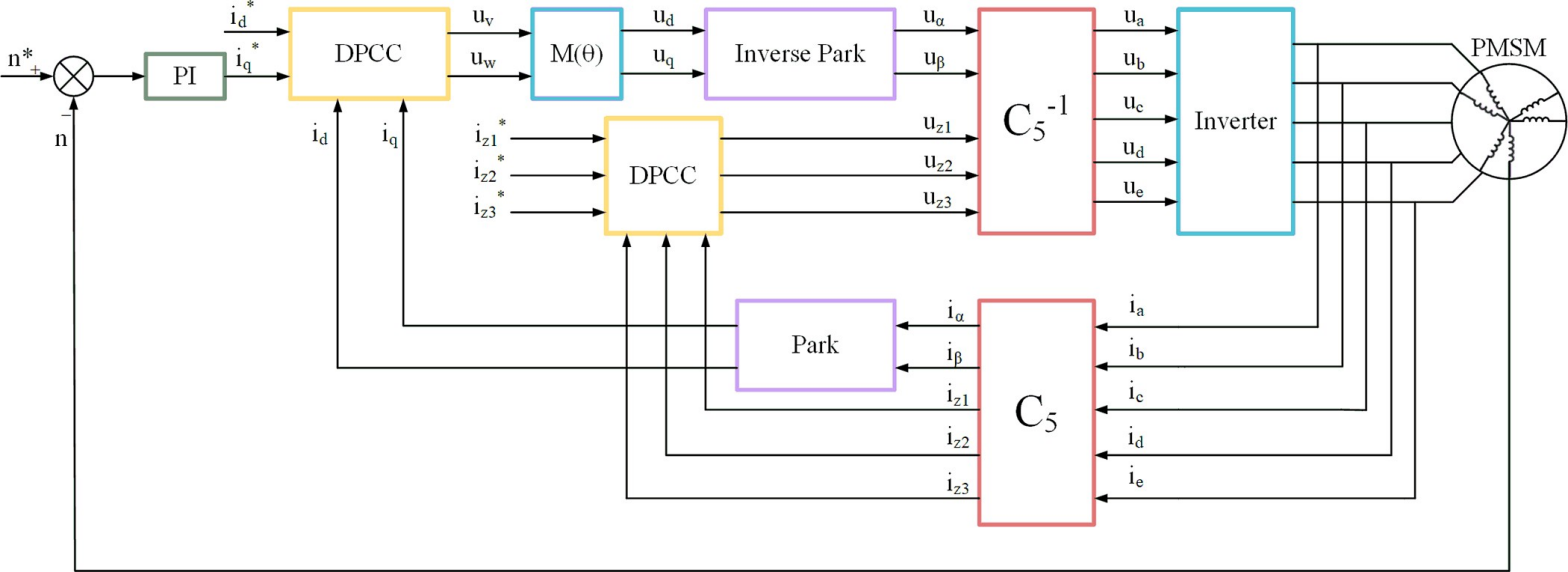
Double-Y phase shift 30° six-phase PMSM model for DPC-FTC in the case of one phase open circuit.

The traditional PI regulator cannot effectively regulate the control system parameters and the response speed is low. The deadbeat current prediction control method is introduced to solve these problems. The PMSM deadbeat current prediction model in the case of one phase open circuit is shown in Fig 3.

For ease of analysis, the current state equation is rewritten in matrix form, and the model is discretized using the forward Euler method to solve for the control voltage at the current moment,:

$$[\begin{array}{c} u_{u}(k)\\ u_{v}(k) \end{array}]=X[\begin{array}{c} i_{d}(k+1)\\ i_{q}(k+1) \end{array}]+Y[\begin{array}{c} i_{d}(k)\\ i_{q}(k) \end{array}]+Z$$ (17)

where:

$$\begin{array}{l} X=\left[\begin{array}{cc} \frac{1.5L_{z}+3L_{d}-0.5\cos(2\theta )L_{z}}{T} & \frac{0.5\sin(2\theta )L_{z}}{T}\\ \frac{0.5\sin(2\theta )L_{z}}{T} & \frac{1.5L_{z}+3L_{q}+0.5\cos(2\theta )L_{z}}{T} \end{array}\right]\\ Y=\left[\begin{array}{cc} 1.5R-0.5\cos(2\theta )R+0.5\sin(2\theta )\omega L_{z}-\frac{1.5L_{z}+3L_{d}-0.5\cos(2\theta )L_{z}}{T} & 0\\ 0.5\sin(2\theta )R+3L_{d}\omega +1.5L_{z}\omega +0.5\cos(2\theta )L_{z}\omega -\frac{0.5\sin(2\theta )L_{z}}{T} & 0 \end{array}\right]\\ \;+\left[\begin{array}{cc} 0 & 0.5\sin(2\theta )R-3L_{q}\omega -1.5L_{z}\omega +0.5\cos(2\theta )L_{z}\omega -\frac{0.5\sin(2\theta )L_{z}}{T}\\ 0 & 1.5R+0.5\cos(2\theta )R-0.5\sin(2\theta )\omega L_{z}-\frac{1.5L_{z}+3L_{q}+0.5\cos(2\theta )L_{z}}{T} \end{array}\right]\\ Z=\left[\begin{array}{c} 0\\ \omega \varphi_{f} \end{array}\right] \end{array}$$ (18)

*i*_*d*_ (*k*)and *i*_*q*_ (*k*) are the current feedback values at the moment of kT sampling, such that *i*_d_*(*k*) = *i*_*d*_ (*k*+1) and *i*_q_*(*k*) = *i*_*q*_(*k*+1). By considering the reference current given as a known input, the reference voltage value at the current moment is computed as:

$$[\begin{array}{c} u_{u}(k)\\ u_{v}(k) \end{array}]=X[\begin{array}{c} i_{d}^{*}(k)\\ i_{q}^{*}(k) \end{array}]+Y[\begin{array}{c} i_{d}(k)\\ i_{q}(k) \end{array}]+Z$$ (19)

The motor model used in this paper has a small *L*_z_, which can be ignored for ease of calculation and stability analysis. For the current prediction model, the control system q-axis voltage equation is given by:

$$\begin{array}{l} u_{v}(k)=\left[(1.5+0.5\cos(2\theta ))R-\frac{3L}{T}\right]i_{q}(k)+\frac{3L}{T}i_{q}(k+1)\\ \;+[0.5\sin(2\theta )R+3L\omega (k)]i_{q}(k)+\omega (k)\varphi_{f} \end{array}$$ (20)

It can be deduced from Eq (20) that the performance of the conventional deadbeat current prediction controller is affected by the motor parameters (in particular, the inductance parameters). If the controller parameters significantly differ from the actual six-phase PMSM parameters, problems such as current distortion and unstable torque can be caused. When the motor inductance and magnetic chain change, the perturbed voltage equation can be rewritten as:

$$\begin{array}{l} u_{v}^{\prime }(k)=\left[(1.5+0.5\cos(2\theta ))R_{0}-\frac{3L_{0}}{T}\right]i_{q}(k)+\frac{3L_{0}}{T}i_{q}^{\prime }(k+1)\\ \;+[0.5\sin(2\theta )R_{0}+3L_{0}\omega (k)]i_{q}(k)+\omega (k)\varphi_{f0} \end{array}$$ (21)

Where *R*_0_, *L*_0_, and *φ*_*f*0_ are respectively the estimated values of the resistance, inductance, and chain parameters used in the current prediction module, *u*_*v*_^ʹ^(*k*) is the cross-axis voltage reference, and *i*_*q*_^ʹ^(*k*+1) is the cross-axis current reference.

When the motor parameters are perturbed, if it is guaranteed that *u*_*v*_(*k*) and *u*_*v*_^ʹ^(*k*) are equal and the sampling period T is sufficiently small, the relationship between the actual current *i*_*q*_(*k*+1) and the reference current *i*_*q*_^ʹ^(*k*+1) is simplified as:

$$\left(3L_{q0}-3L_{q})i_{q}(k)+3L_{q}i_{q}(k+1)=3L_{q0}i_{q}^{\prime }(k+1\right)$$ (22)

The discrete transfer functions of the actual and reference currents are obtained by applying a Z-transform to Eq (22):

$$\frac{i_{q}(z)}{i_{q}^{\prime }(z)}=\frac{zL_{q0}}{zL_{q}-(L_{q}-L_{q0})}$$ (23)

Similar to the analysis of Eq (23), a straight axis current discrete transfer function, which only has a pole *z* = 1 - (*L*_*q*0_ / *L*_*q*_), can be obtained. According to the control theory, if all the poles are in the unit circle, then the system tends to be stable. Based on the stability analysis of the system, the stability conditions are given by:

$$\{\begin{array}{c} 0<L_{d0}<2L_{d}\\ 0<L_{q0}<2L_{q} \end{array}$$ (24)

It can be concluded that the inductance parameters of the six-phase PMSM fault-tolerant control system significantly affect the stability of the system. When the inductance parameter of the DPCC module is less than 2 times the actual inductance value, the actual and reference current discrete transfer functions converge and the system tends to be stable. When the inductance of the DPCC module is greater than 2 times the actual inductance, the current discrete transfer function diverges and the system tends to be unstable.

### 3.2 Deadbeat current predictive fault-tolerant control with weight coefficient

In response to the instability of the drive system caused by parameter perturbation, this paper improves the traditional deadbeat current prediction model by introducing weight coefficients (α and β) to correct the current error caused by parameter perturbation. This allows the drive system to be less sensitive to parameter perturbation and to have a wider stability margin. The weight coefficients are used to split and optimize the d and q axis currents at the kT moment. The specific optimization algorithm is given by:

$$\{\begin{array}{c} i_{d}(k)=\alpha i_{d}^{*}(k)+\beta i_{d}(k)\\ i_{q}(k)=\alpha i_{q}^{*}(k)+\beta i_{q}(k) \end{array}$$ (25)

The voltage equation under constant desired voltage before and after inductor parameter perturbation is obtained by substituting Eq (25) into Eq (20) and (21), and the controller sampling period is considered small enough, which simplifies the relational equation for the optimized DPCC controller.

$$\begin{array}{l} \left(1.5L_{z}+0.5\cos(2\theta )L_{z}+3L_{q})i_{q}(k+1\right)\\ +[\beta (1.5L_{z0}+0.5\cos(2\theta )L_{z0}+3L_{q0})-(1.5L_{z}+0.5\cos(2\theta )L_{z}+3L_{q})]i_{q}(k)\\ =0.5\sin(2\theta )L_{z}i_{d}(k)-0.5\sin(2\theta )L_{z}i_{d}(k+1)-\alpha (1.5L_{z0}+0.5\cos(2\theta )L_{z0}+3L_{q0})i_{q}^{\prime }(k)\\ +(1.5L_{z0}+0.5\cos(2\theta )L_{z0}+3L_{q0})i_{q}^{\prime }(k+1)\\ +0.5\sin(2\theta )L_{z0}(\alpha i_{d}^{\prime }(k)+\beta i_{d}(k)) \end{array}$$ (26)

where the weighting coefficients 0 < *α* < 1 and 0 < *β* < 1 satisfy the constraint *α* + *β* = 1.

It is considered that the used motor model has negligible leakage inductance, and thus the relationship is simplified as:

$$\left(3\beta L_{q0}-3L_{q})i_{q}(k)+3L_{q}i_{q}(k+1)=3L_{q0}i_{q}^{\prime }(k+1)-3\alpha L_{q0}i_{q}^{\prime }(k\right)$$ (27)

Eq (27) is Z-transformed to obtain the discrete transfer function for the intersection axis, and the discrete transfer function for the straight axis is derived in a similar way:

$$\{\begin{array}{c} \frac{i_{d}(z)}{i_{d}^{\prime }(z)}=\frac{zL_{d0}-\alpha L_{d0}}{zL_{d}-(L_{d}-\beta L_{d0})}\\ \frac{i_{q}(z)}{i_{q}^{\prime }(z)}=\frac{zL_{q0}-\alpha L_{q0}}{zL_{q}-(L_{q}-\beta L_{q0})} \end{array}$$ (28)

**Fig 4:**
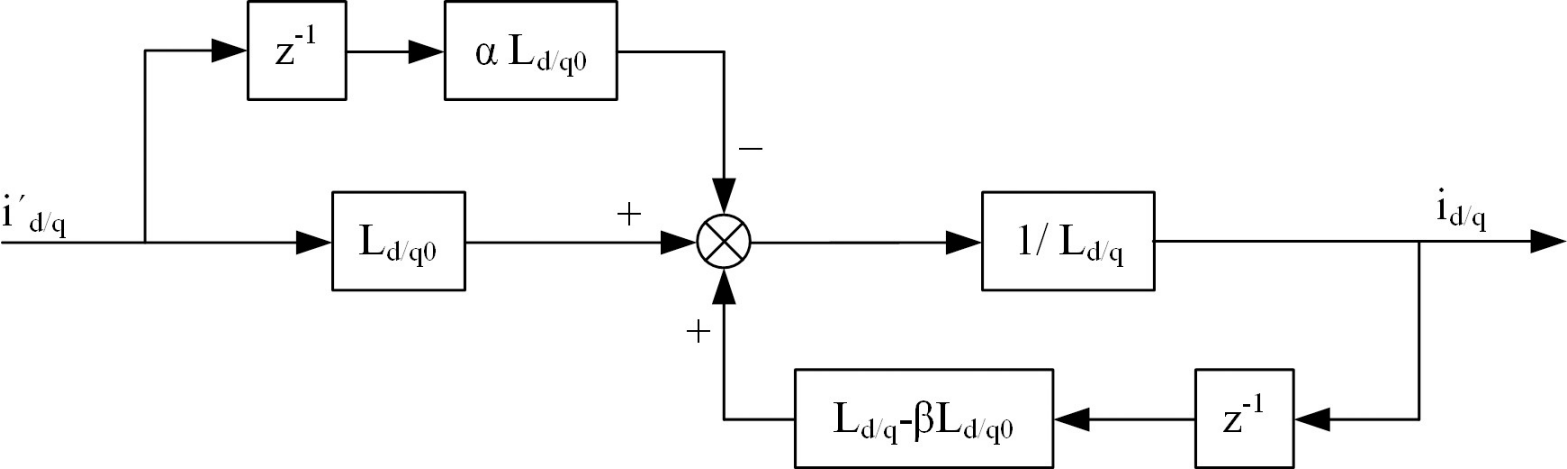
Equivalent model of current predictive fault-tolerant control algorithm before and after parameter perturbation.

The equivalent model before and after the parameter perturbation of the DPC-FTC is shown in Fig 4.

It can be seen from Eq (28) that there is only one pole of the discrete transfer function of the d and q axis current before and after parameter perturbation: *z* = 1 - *β* (*L*_*d*0_ / *L*_*d*_) and *z* = 1 - *β* (*L*_*q*0_ / *L*_*q*_). If all the poles of the discrete transfer function are located within the unit circle of the Z domain, the DPC-FTC driven system tends to be stable, and therefore the system stability condition is given by:

$$\{\begin{array}{c} 0<L_{d0}<\frac{2}{\beta }L_{d}\\ 0<L_{q0}<\frac{2}{\beta }L_{q} \end{array}$$ (29)

**Fig 5:**
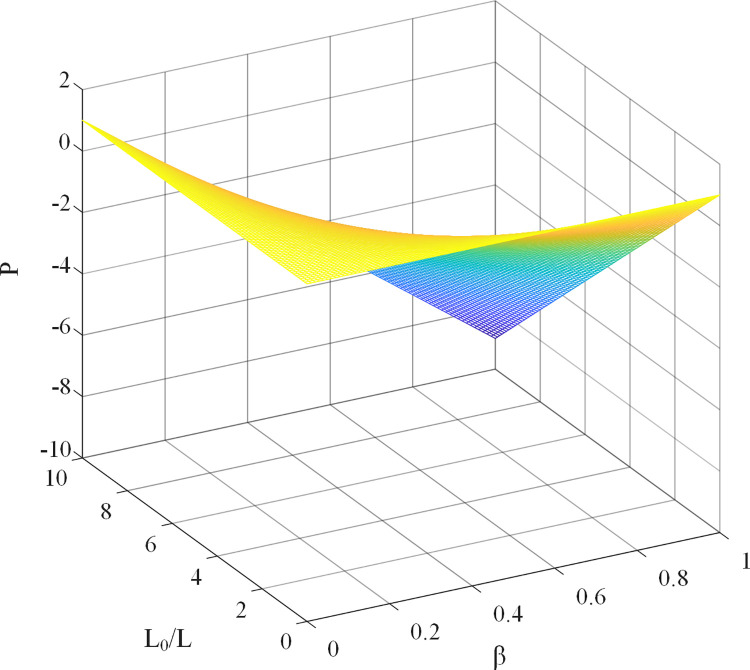
Relationship between the system poles, β, and L0 / L.

β varies between 0 and 1. By combining the system stability conditions of Eq (24) and (29), it can be deduced that the discrete transfer function pole is affected by the value of β. The sensitivity of the system stability to the inductance parameter with the introduction of the weighting coefficient varies with the change of β, and the permissible range of inductance parameter variation when the improved system stabilizes is expanded by a factor of 1/β. As β decreases, the allowable range of inductance error increases when the system tends to be stable. Therefore, the introduction of weight coefficient can reduce the influence of system stability reduction caused by parameter perturbation in the deadbeat current predictive fault-tolerant control algorithm. The relationship between the system pole, β, and L_0_/L is shown in Fig 5.

## 4 Simulation analysis

**Fig 6:**
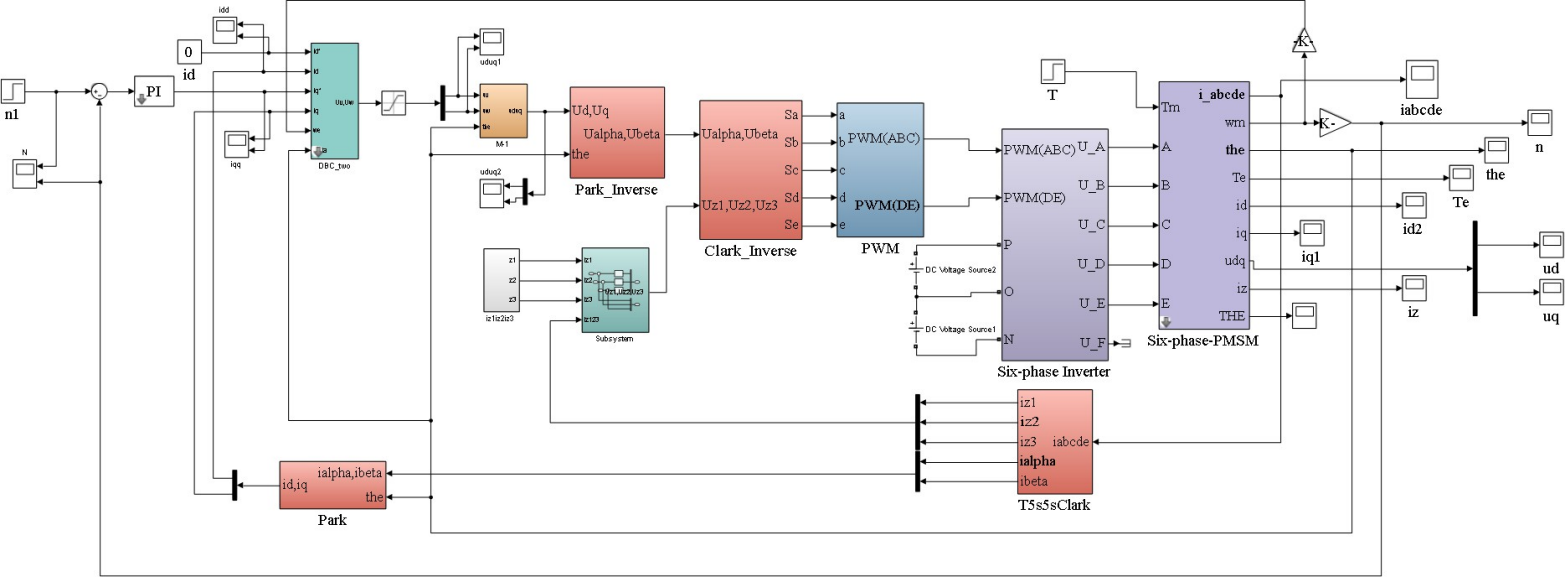
Deadbeat current predictive fault-tolerant control system model in the case of one phase open circuit.

To verify the correctness of the fault-tolerant control theory of deadbeat current prediction with the proposed introduction of weight coefficients, the conventional DPC-FTC and the improved DPC-FTC model are evaluated. They are developed on MATLAB/Simulink for separate experiments. The motor model used in the simulation is a double-Y phase shift 30° six-phase PMSM with the following parameters: *R* = 0.05Ω, *L*_*d*_ = *L*_*q*_ = 0.89mH, *ψ*_*f*_ = 0.58Wb and the number of pole pairs is 3. The given speed is 500 r/min and the weighting coefficients α = 0.5 and β = 0.5 are set to simulate the parameter mismatch by changing the ratio of controller inductance parameters to motor inductance parameters, and the simulations are carried out separately. The DPC-FTC simulation model is shown in Fig 6.

**Fig 7:**
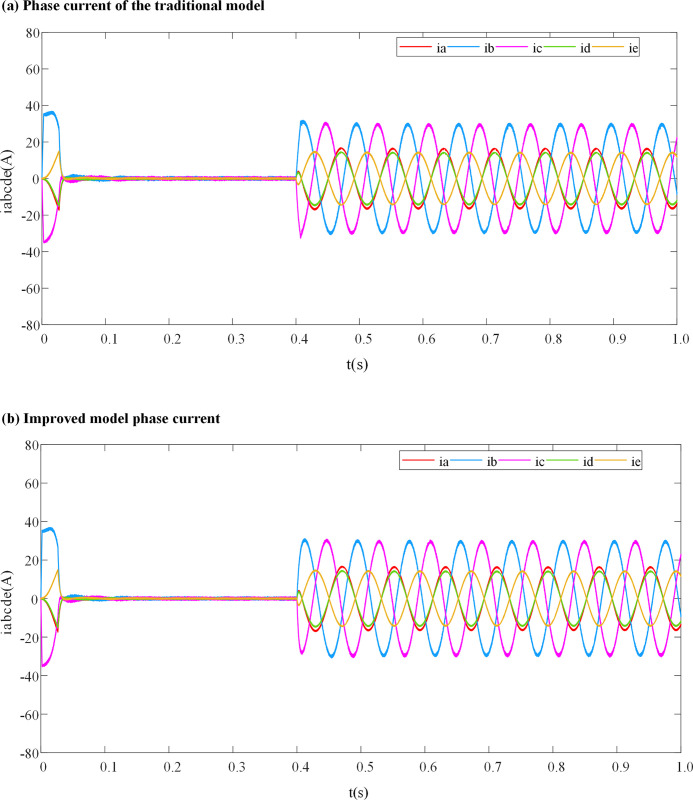
Phase current waveform when L_0_ = L. (a) Phase current of the traditional model; (b) Improved model phase current.

Fig 7(A) and 7(B) correspond to the simulated phase currents of the traditional DPC-FTC model and the DPC-FTC model after introducing the weight coefficient. The motor runs at an initial speed of 500 r/min without load, 80 N⸱m torque is added at 0.4 s, and the inductance parameters of the controller are consistent with the inductance parameters of the motor.

It can be seen from Fig 7 that when the inductance parameters of the DPCC module are consistent with the actual inductance parameters of the motor, there is no significant difference between the remaining phase currents before and after the introduction of the weight coefficient.

**Fig 8:**
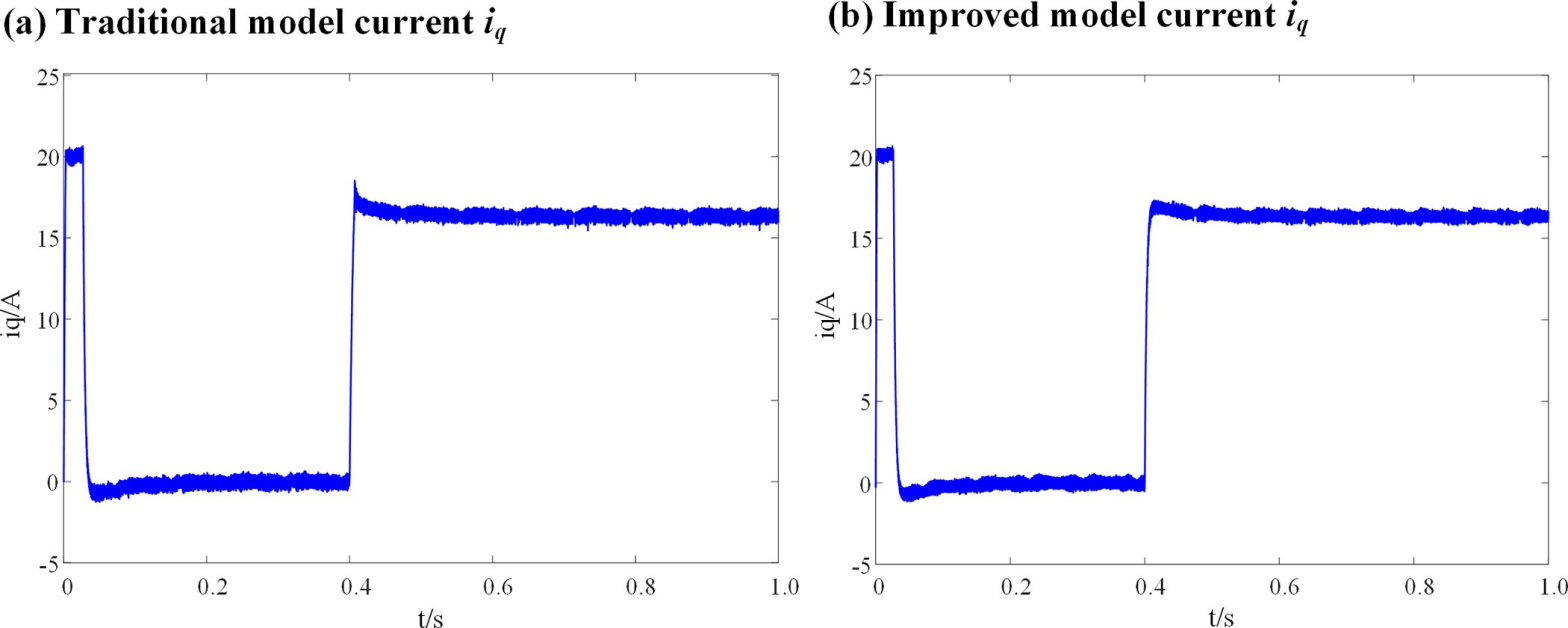
Current *i*_*q*_ waveform at L_0_ = L. (a) Traditional model current *i*_*q*_; (b) Improved model current *i*_*q*_.

Fig 8 show a comparison between the current (*i*_*q*_) waveforms of the conventional deadbeat fault-tolerant model and the improved deadbeat fault-tolerant model with the introduction of the weighting coefficient, when the inductance parameters of the DPCC module are consistent with the actual inductance parameters of the motor.

It can be seen from Fig 8, that when the inductance parameters of the DPCC module are consistent with the actual inductance parameters of the motor, there is no significant difference in the current (*i*_*d*_) before and after the introduction of the weighting coefficient.

**Fig 9:**
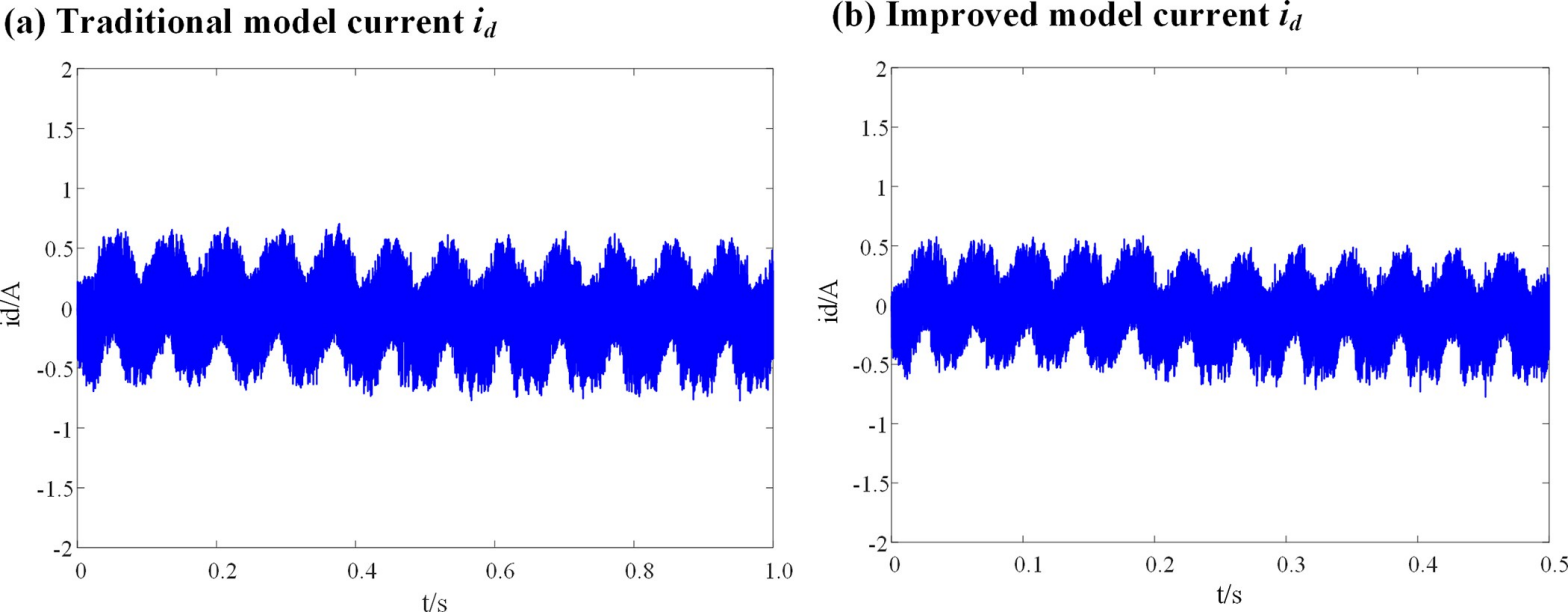
Current *i*_*d*_ waveform at L_0_ = L. (a) Traditional model current *i*_*d*_; (b) Improved model current *i*_*d*_.

Fig 9 show a comparison between the current (*i*_*d*_) waveforms of the conventional deadbeat fault-tolerant model and the improved deadbeat fault-tolerant model with the introduction of the weighting coefficient, when the inductance parameters of the DPCC module are consistent with the actual inductance parameters of the motor.

**Fig 10:**
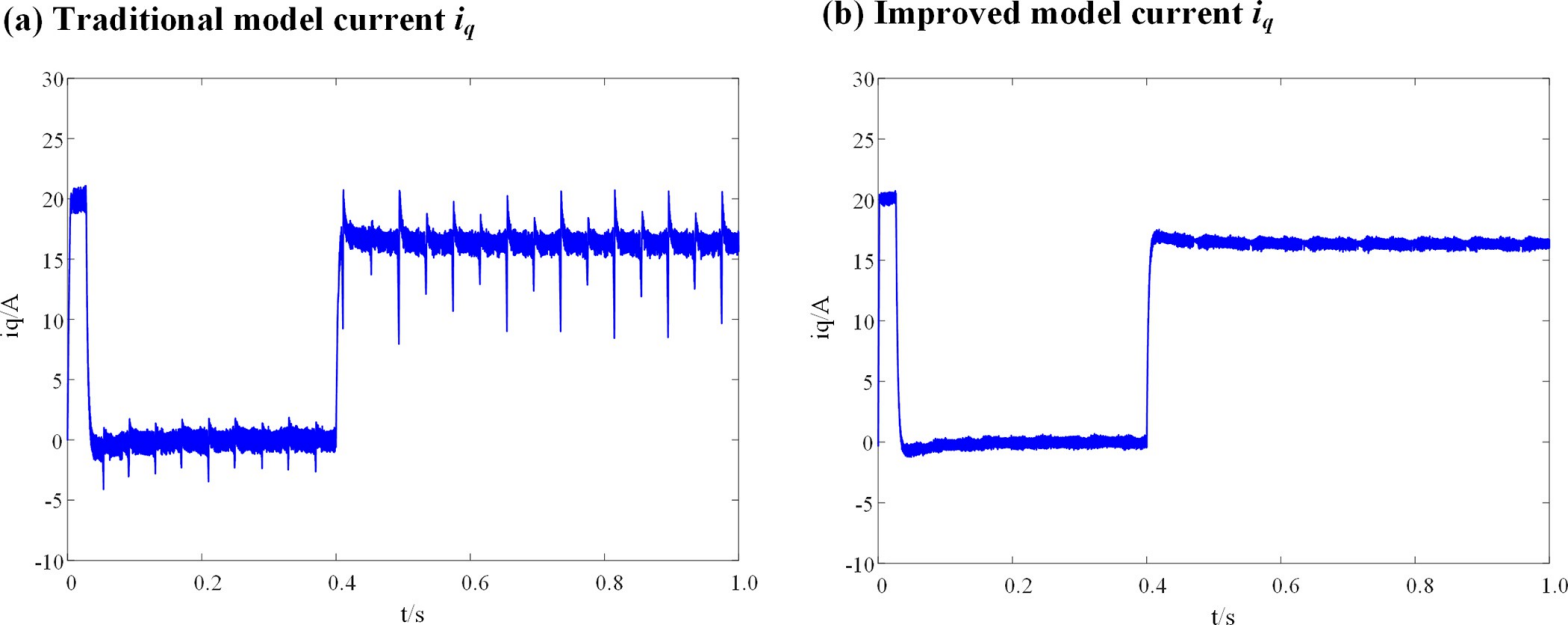
Current *i*_*q*_ waveform at L_0_ = 2L. (a) Traditional model current *i*_*q*_; (b) Improved model current *i*_*q*_.

It can be seen from Fig 9, that when the inductance parameters of the DPCC module are consistent with the actual inductance parameters of the motor, there is no significant difference in the current (*i*_*q*_) before and after the introduction of the weighting coefficient. When the motor is in fault-tolerant control operation, the *i*_*d*_ current vibration range of the traditional DPC-FTC model is between -0.7 A and 0.7 A, and that of the DPC-FTC model after introducing the weight coefficient is between -0.55 A and 0.54 A. Fig 10 show the simulation results of the *i*_*q*_ current before and after the introduction of the weight coefficient when the inductance parameter of the DPCC module is two times that of the motor.

**Fig 11:**
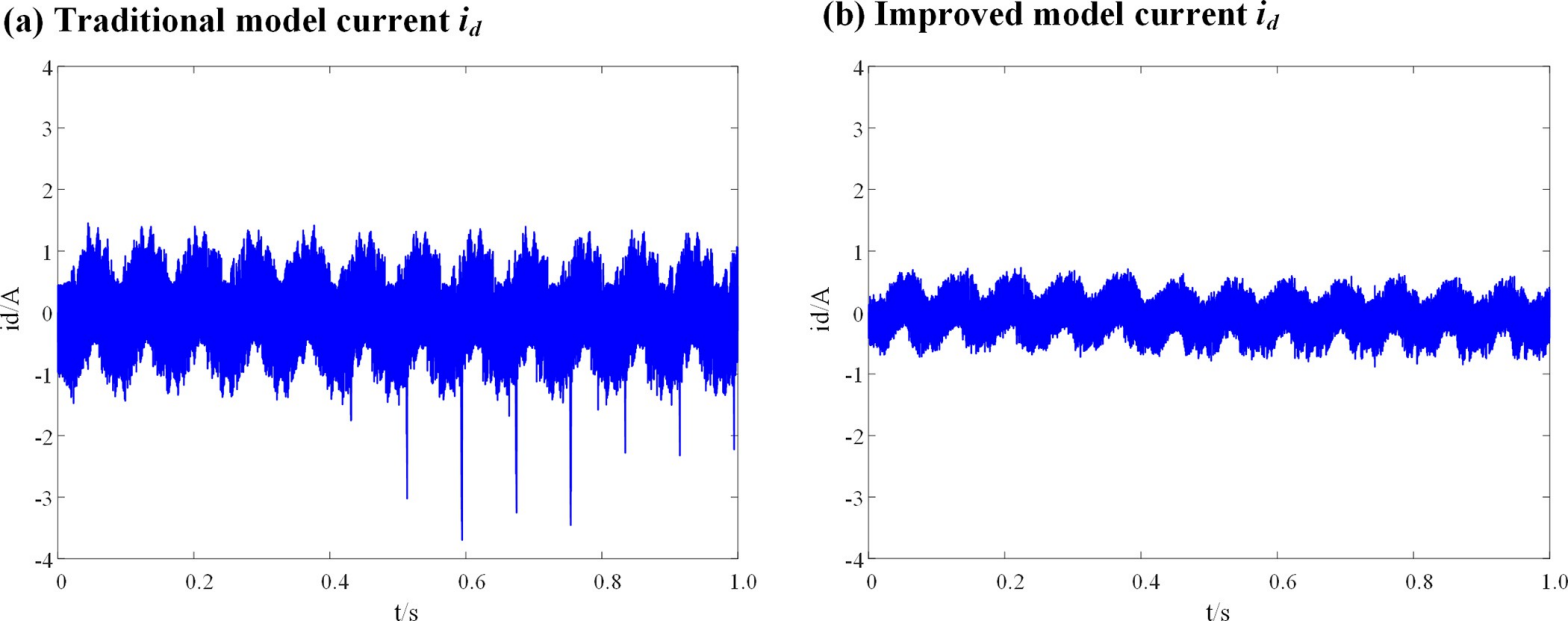
Current *i*_*d*_ waveform at L_0_ = 2L. (a) Traditional model current *i*_*d*_; (b) Improved model current *i*_*d*_.

The simulation results of the id current for the DPC-FTC model are presented in Fig 11, both before and after the introduction of the weight coefficient when the inductance parameter of the DPCC module is two times that of the motor.

**Fig 12:**
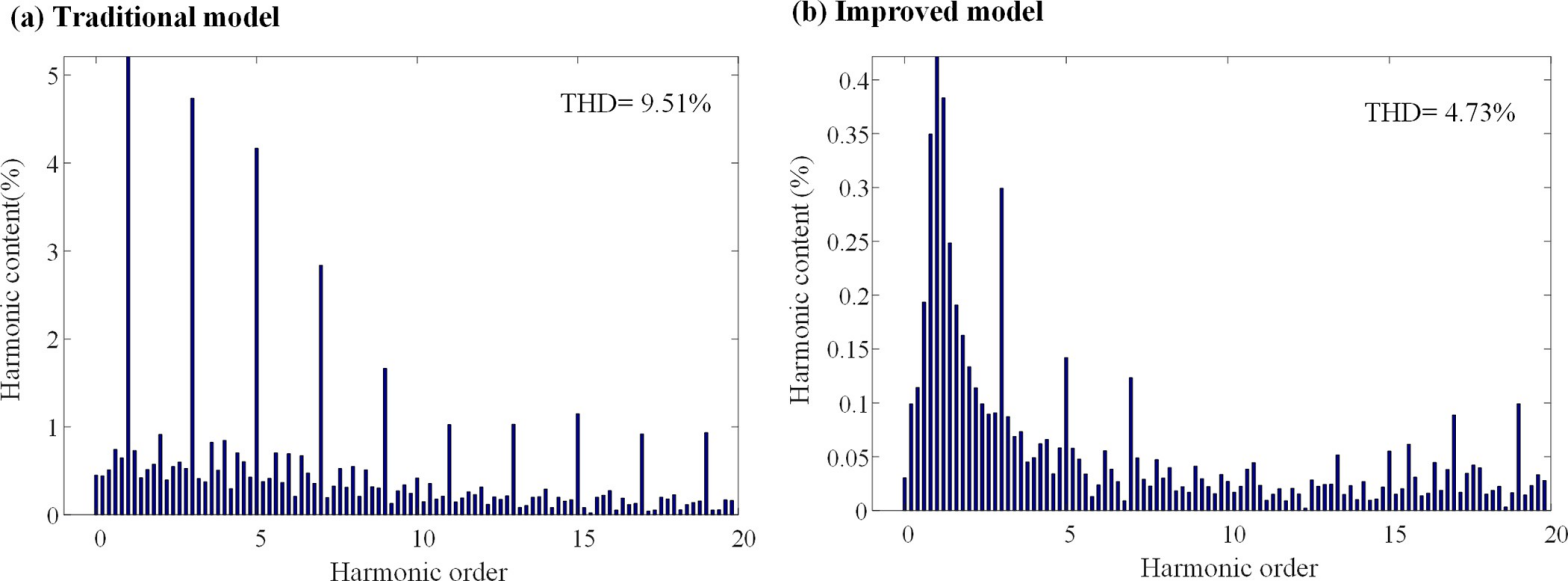
Stator current spectrum analysis at L0 = 2L. (a) Traditional model; (b) Improved model.

**Fig 13:**
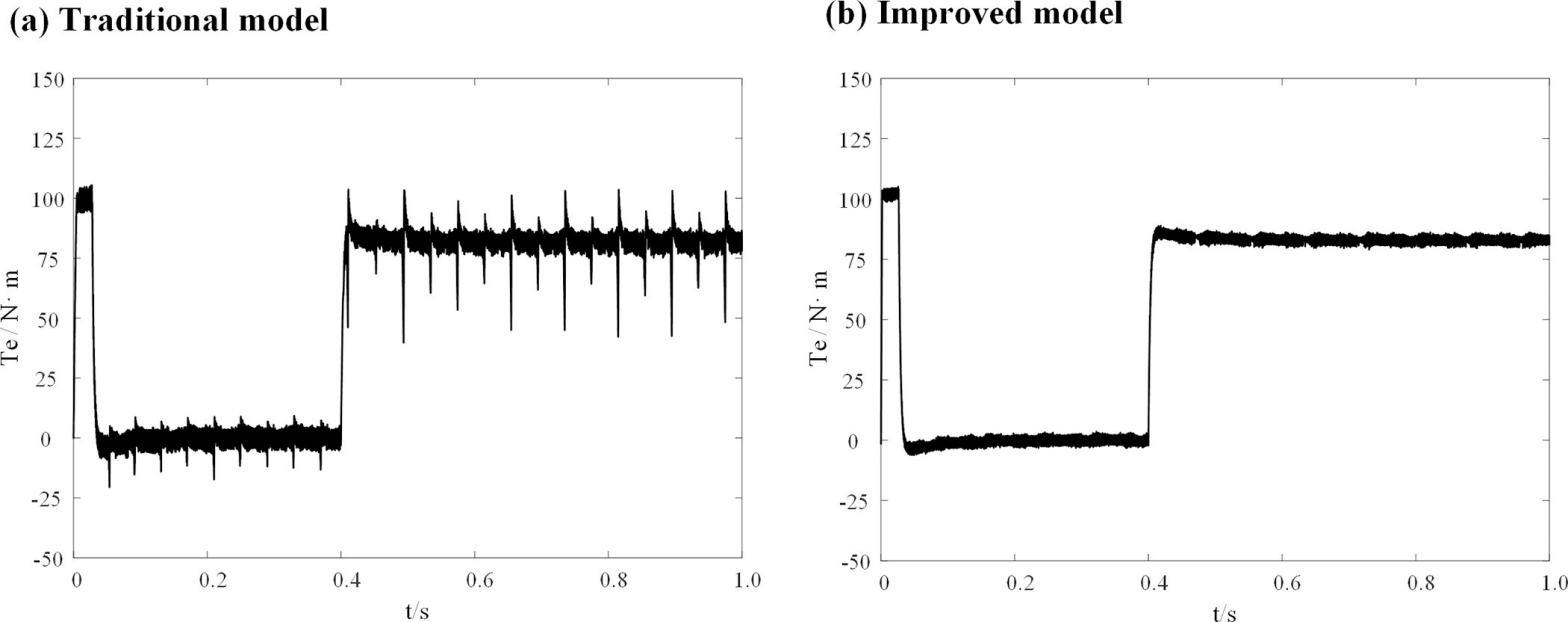
Torque waveform at L0 = 2L. (a) Traditional model; (b) Improved model.

When the inductance parameter of the controller is greater than two times that of the motor, the system will be unstable, as mentioned in Section 3. Figs 10 and 11 show that when the controller inductance parameter is two times the motor inductance, the traditional DPC-FTC model motor steady-state operating current significantly oscillates, and the operating *i*_*d*_ vibration is between -1.45 A and 1.45 A, while for the model after the introduction of the weighting coefficient, the *i*_*q*_ current oscillation is significantly improved and its oscillation range is reduced to approximately [-0.8–0.75 A]. In addition, an analysis is conducted on the total harmonic distortion rate (THD) of the stator current for both the conventional DPC-FTC model and the improved DPC-FTC model. The stator current spectrum analysis is presented in Fig 12. The THD for the conventional model is found to be 9.51%, whereas for the improved model, it is reduced to 4.73%. Furthermore, the torque curve is shown in Fig 13, indicating that the introduction of the weighting factor effectively suppresses stator current distortion and reduces torque pulsation.

**Fig 14:**
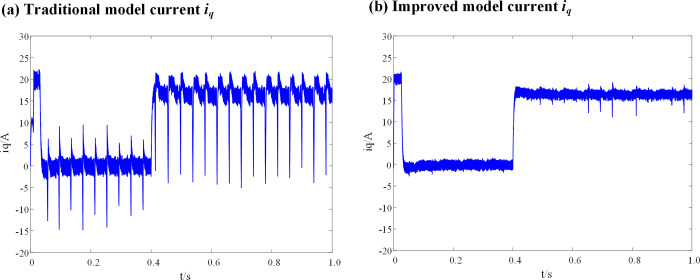
Current *i*_*q*_ waveform at L_0_ = 4L. (a) Traditional model current *i*_*q*_; (b) Improved model current *i*_*q*_.

**Fig 15:**
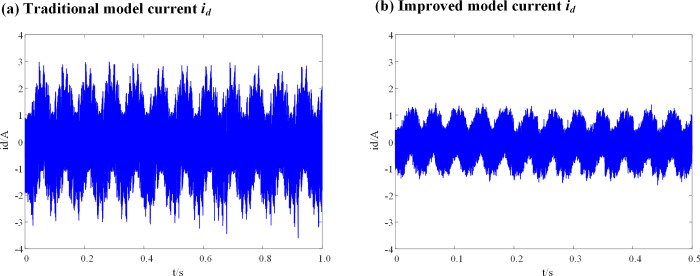
Current *i*_*d*_ waveform at L_0_ = 4L. (a) Traditional model current *i*_*d*_; (b) Improved model current *i*_*d*_.

Considering the errors in the initial setting of the controller parameters and the mismatch of the motor inductance under the maximum operating conditions, a 4-fold mismatch is set. Figs 14 and 15 show a simulation current comparison between the traditional DPC-FTC model and the improved DPC-FTC model after introducing the weight coefficient, when the controller inductance parameters are four times the motor inductance parameters.

It can be seen that when the inductance parameter of the controller is 4 times the inductance of the motor, the current of the traditional DPC-FTC model oscillates violently, and the operating *i*_*d*_ vibration is between -3.7A and 3 A, while for the model after the introduction of the weighting coefficient, the current *i*_*d*_ and *i*_*q*_ oscillations of the DPC-FTC model are significantly improved and the operating *i*_*d*_ current is reduced to approximately [-1.7–1.45 A].

**Fig 16:**
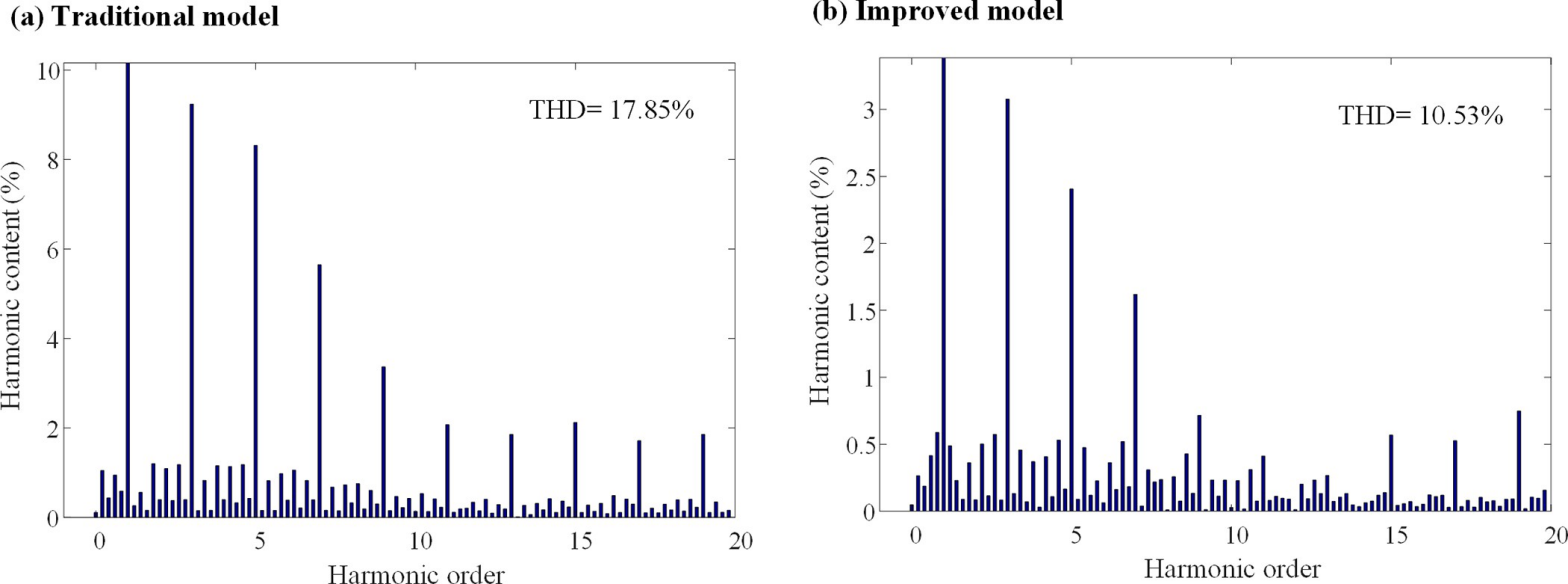
Stator current spectrum analysis at L0 = 4L. (a) Traditional model; (b) Improved model.

**Fig 17:**
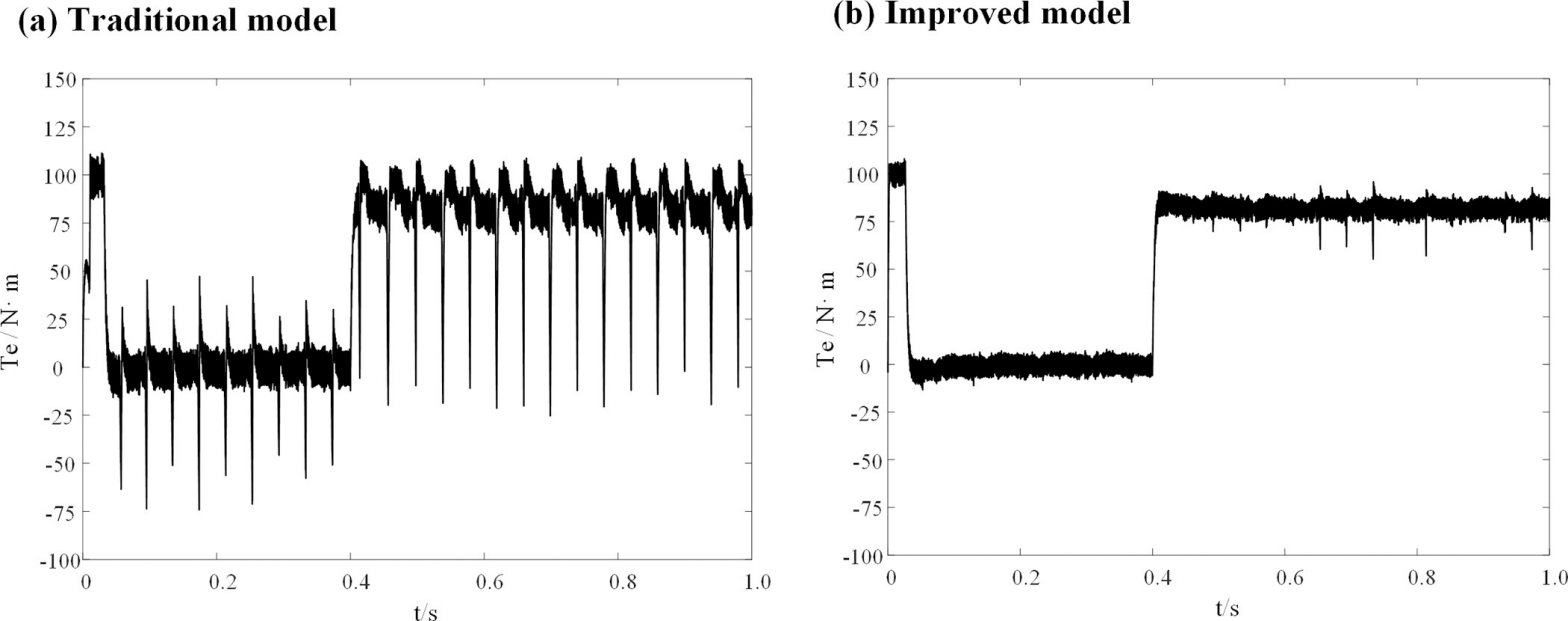
Torque waveform at L0 = 4L. (a) Traditional model; (b) Improved model.

To compare the performance of the conventional DPC-FTC model with the improved DPC-FTC model, the stator current spectrum analysis is presented in Fig 16, revealing the total harmonic distortion rate before and after the improvement as 17.85% and 10.53%, respectively. Furthermore, the torque curve is shown in Fig 17, which indicates that by introducing the weighting factor, the output current harmonic components are greatly suppressed, leading to a reduction in torque pulsation when the inductance parameter of the controller is four times the motor inductance.

The obtained results prove the feasibility of the proposed improved DPC-FTC algorithm with weight coefficient. The improved DPC-FTC strategy can perform fault-tolerant and stable operation when single-phase phase-missing fault occurs in six-phase PMSM with double Y phase shift of 30°, and it effectively reduces the influence of the inductance parameter perturbation on the system stability.

## 5 Experimental verification

**Fig 18:**
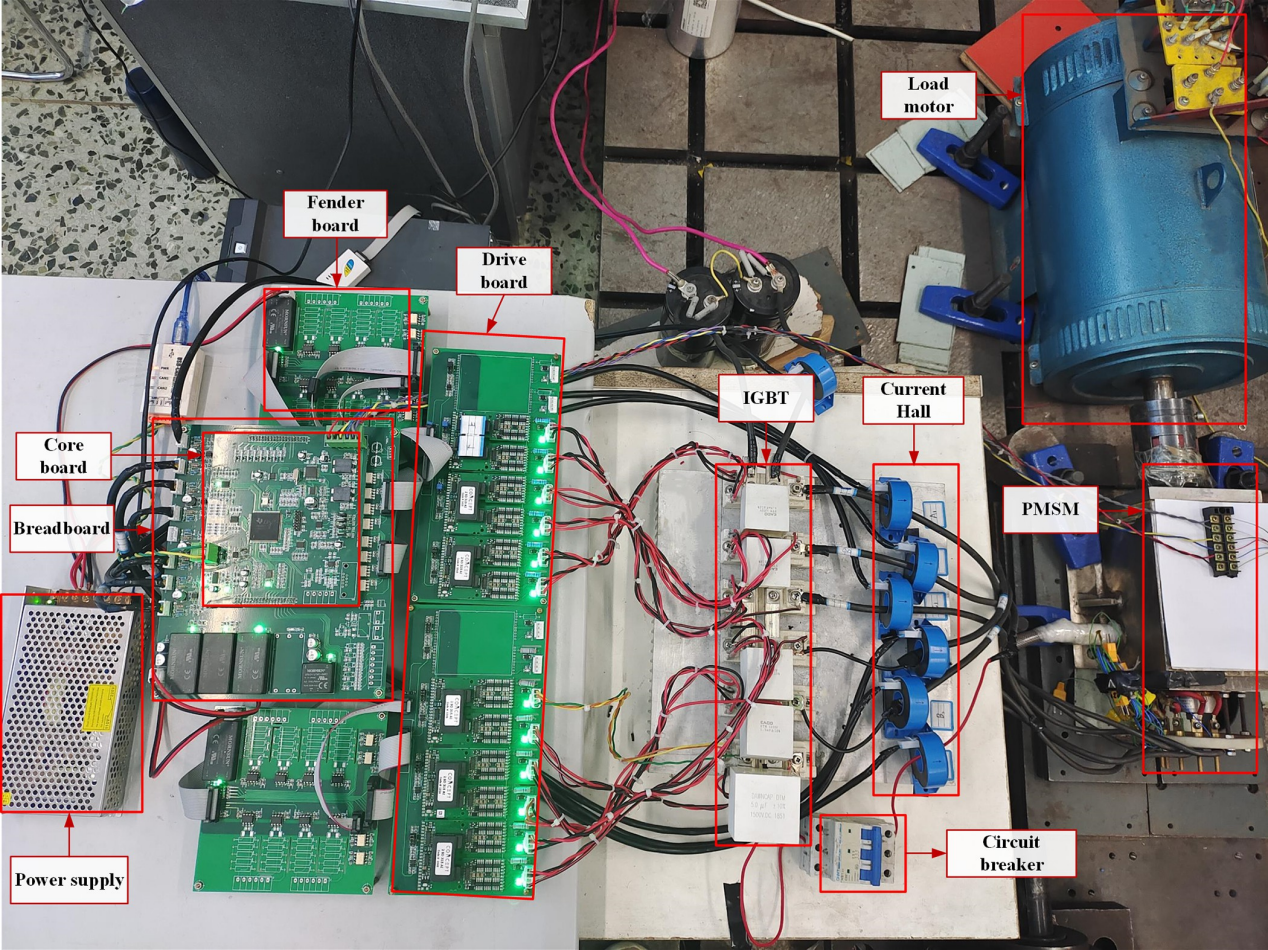
Experimental platform.

To further verify the correctness and feasibility of the improved DPC-FTC algorithm proposed above, an experimental platform based on TMS32028335 chip is built with double-Y phase shift 30° six-phase PMSM as the research object. The DPC-FTC experimental platform is shown in Fig 18. It includes a core board, an analog board, an open circuit protection board, a drive board, a power supply, a drive motor, and a load motor. In the experiment, the dual Y phase shift 30° six-phase PMSM is used to perform the load operation of the towed synchronous generator. The change of the direct and quadrature axis inductance under the motor working condition is first measured and fitted. The air switch is then used to disconnect the single-phase winding of the motor from the inverter in order to simulate the phase loss fault of the motor. The motor parameters are shown in Table 1.

**Table 1:** Motor parameters.

Parameter	Value
Nominal power	10kw
Rated current	19.92A
Rated speed	1000r/min
Rated voltage	311V
Rated torque	90N⸱m
Sampling time	100μs
Switching frequency	10kHz
Stator resistance	0.22Ω
D-axis inductance	0.89mH
Q-axis inductance	0.89mH
Number of pole-pairs	3

**Fig 19:**
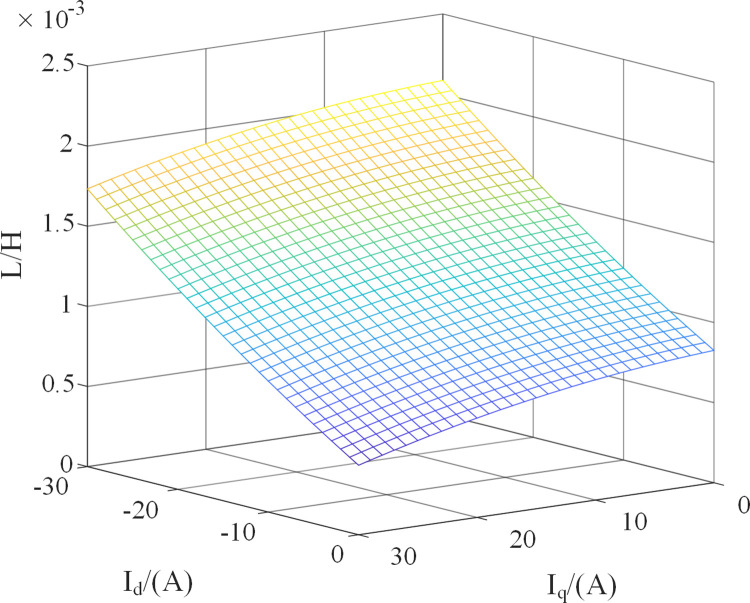
Equivalent inductance variation fitting of PMSM.

The inductance change of the motor used in the experiment is tested and fitted while ignoring the influence of the temperature. The inductance change is shown in Fig 19.

**Fig 20:**
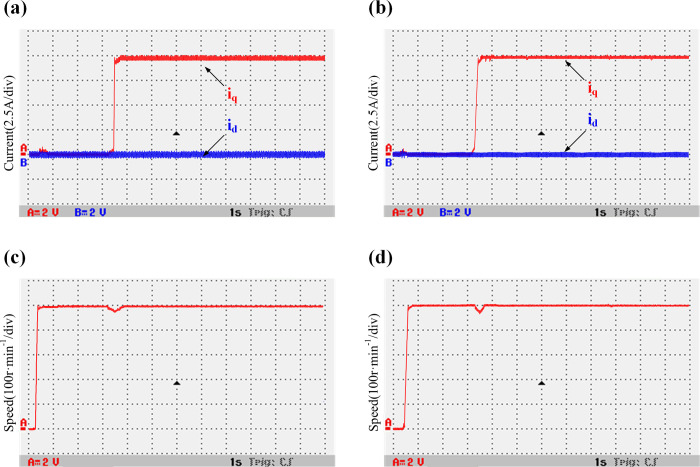
Comparison between the waveforms of the variable torque current and speed, when the inductance parameters of the traditional and improved models are consistent. (a) Traditional model current *i*_*d/q*_; (b) Improved model current *i*_*d/q*_; (c) Traditional model speed *n*; (d) Improved model speed *n*.

**Fig 21:**
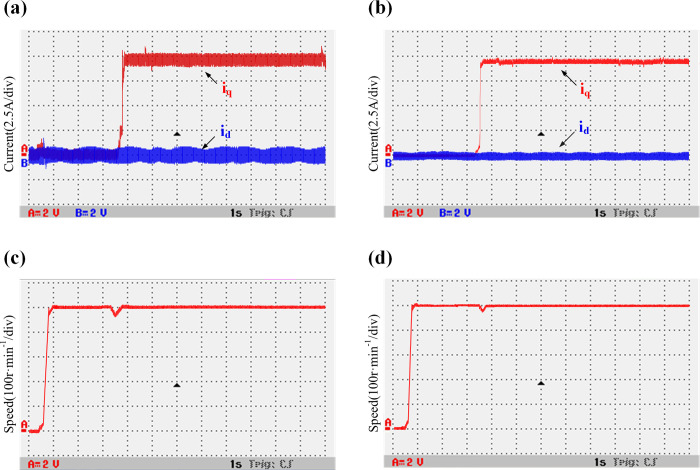
Comparison between the waveforms of the variable torque current and speed, when a two times inductance mismatch exists between the traditional and improved models. (a) Traditional model current *i*_*d/q*_; (b) Improved model current *i*_*d/q*_; (c) Traditional model speed *n*; (d) Improved model speed *n*.

The double-Y phase shift 30° six-phase PMSM starts at an initial speed of 500 r/min and suddenly adds 50 N⸱m load torque in 3.5 s. The motor’s F-phase winding connection is disconnected by an air switch to perform the F-phase fault-tolerant operation of the motor. The conventional algorithm and the improved algorithm with the introduction of weighting coefficients (α = 0.5 and β = 0.5) are evaluated and compared. In the experiment, the current is converted to voltage equivalent measurement. A 2.5 A current value is equivalent to a 2 V voltage value. Fig 20(A) and 20(C) show *i*_*d*_, *i*_*q*_, and speed waveforms of the traditional deadbeat predictive current fault-tolerant control when the controller inductance is consistent with the motor inductance parameters. Fig 20(B) and 20(d) show the current *i*_*d*_, *i*_*q*_ and speed waveforms of the improved deadbeat predictive current fault-tolerant control when the controller inductance is consistent with the motor inductance parameters. It can be observed that the speed of the motor is stable during fault-tolerant operation. In the case of consistent inductance parameters, the *i*_*d*_ oscillation range of the traditional algorithm is [-0.45–0.45 A], and that of the improved algorithm is [-0.4–0.35 A]. The current waveform is not much different. Fig 21(A) and 21(C) show *i*_*d*_, *i*_*q*_, and speed waveforms of the traditional deadbeat predictive current fault-tolerant control when the controller inductance and the motor inductance parameters have a two times mismatch. Fig 21(B) and 21(D) show the current *i*_*d*_, *i*_*q*_ and speed waveforms of the improved deadbeat current predictive fault-tolerant control when the controller inductance and the motor inductance parameters have a two times mismatch. It can be deduced that the current oscillation is obvious. The *i*_*d*_ oscillation range of the traditional model is [-1–0.95 A], and that of the improved model is [-0.55–0.45 A]. It can be clearly seen that the current oscillation is improved.

The motor starts at an initial speed of 250 r/min. A 30 N⸱m load is then added, and the speed is set to 500 r/min at 5 s. The traditional DPC-FTC and the improved DPC-FTC are evaluated and compare.

**Fig 22:**
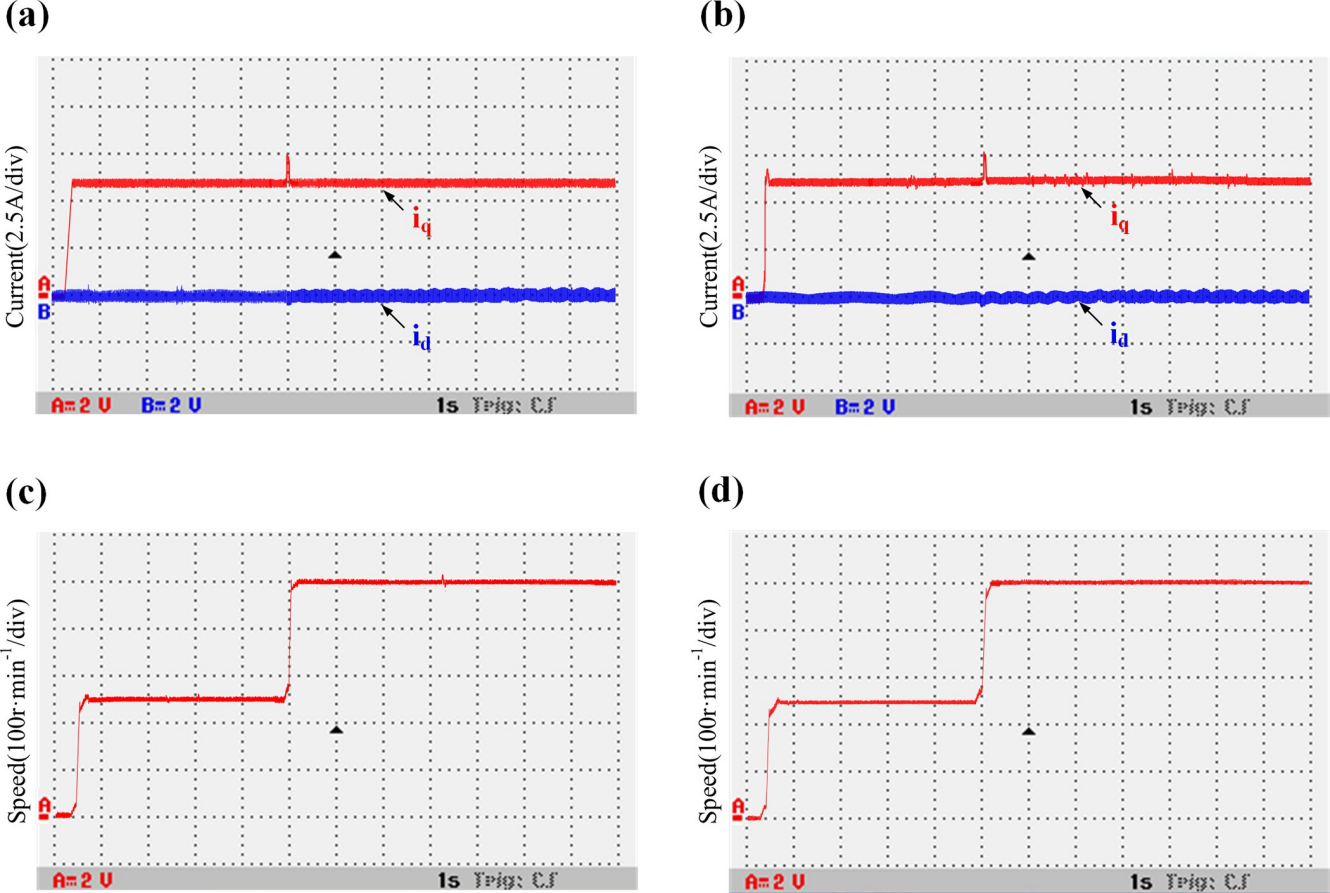
Comparison between the waveforms of the variable speed current and speed when the inductance parameters of the traditional and improved models are consistent. (a) Traditional model current *i*_*d/q*_; (b) Improved model current *i*_*d/q*_; (c) Traditional model speed *n*; (d) Improved model speed *n*.

**Fig 23:**
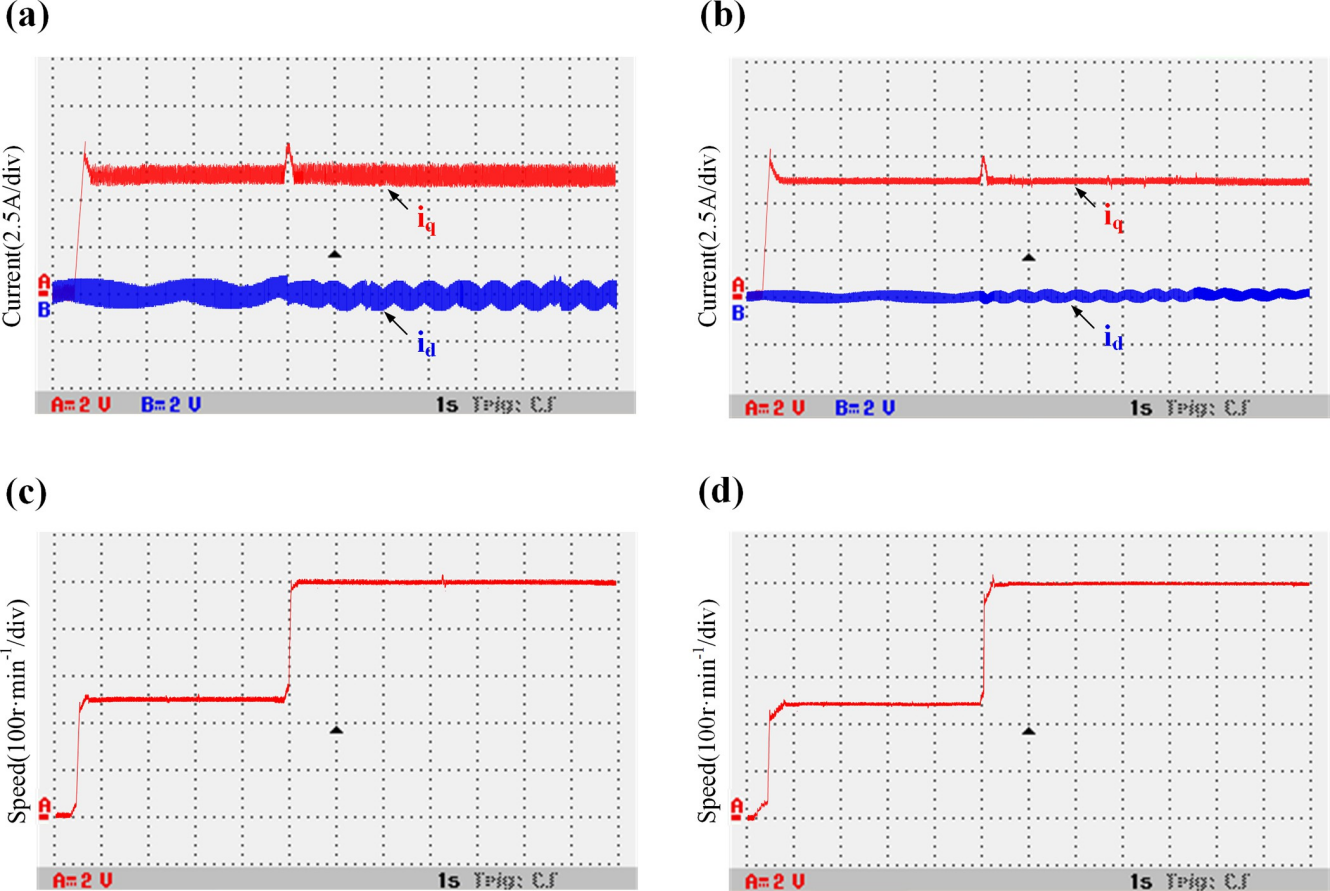
Comparison between the waveforms of the variable speed current and speed of the traditional and improved models when the inductance mismatch is two times. (a) Traditional model current *i*_*d/q*_; (b) Improved model current *i*_*d/q*_; (c) Traditional model speed *n*; (d) Improved model speed *n*.

Fig 22 shows a comparison between the waveforms of *i*_*d*_, *i*_*q*_, and speed when the parameters of the controller inductance and motor inductance are consistent. Fig 23 shows the comparison between the waveforms of *i*_*d*_, *i*_*q*_, and speed when the parameters of controller inductance and motor inductance have a two times mismatch. It can be seen that when the inductance parameters are consistent, the speed of the motor is stable during fault-tolerant operation. The current *i*_*d*_ oscillation range of the traditional model is [-0.5–0.45 A], and the current *i*_*d*_ oscillation range of the improved model is [-0.45–0.45A], and the current is not much different. When the controller inductance and the motor inductance parameters have a two times mismatch, the speed is stable during fault-tolerant operation, the traditional model current *i*_*d*_ oscillation is obvious, the oscillation range is [-1–0.95A], the improved model current oscillation range is [-0.45–0.5A]. It can be clearly seen that the current oscillation is improved.

Table 2 shows a comparison of the steady-state current error between the two algorithms when the inductor parameters are mismatched.

**Table 2:** Comparison of the two algorithms for the d-axis current static error when the inductor parameters are mismatched.

Operational status	Inductance mismatch	Current static difference/A		Static difference reduction ratio/%
		Traditional algorithms	Improved algorithms	
**Variable torque**	L_0_ = L	0.9	0.75	16.67
	L_0_ = 2L	1.95	1	48.72
**Variable speed**	L_0_ = L	0.9	0.95	5.26
	L_0_ = 2L	1.95	0.95	51.28

In summary, the feasibility of the proposed improved deadbeat current predictive fault-tolerant control algorithm is proved. Compared with the traditional DPC-FTC model, the improved DPC-FTC model significantly increases the inductance perturbation range of the double-Y phase shift 30° six-phase PMSM stable fault-tolerant operation, effectively suppresses current oscillations and reduces current static differences.

## 6 Conclusion

In this paper, a six-phase PMSM with double-Y phase shift 30° is tackled, and an improved DPC-FTC strategy is proposed. The mathematical model of a six-phase motor in normal operation is first analyzed. The dimensional reduction mathematical model of the motor in single-phase fault operation is then reconstructed according to its spatial distribution of windings and vector decoupling transformation. The dimensional reduction voltage equation is derived and quadratically transformed to perform complete decoupling control in order to conduct fault-tolerant control. Considering the inadequate dynamic performance of the current loop when utilizing a PI regulator, the deadbeat current prediction algorithm is employed in lieu of traditional PI regulation for control, and the expected voltage is predicted by the motor current feedback value and reference value to improve the fault-tolerant control performance. The voltge equation after inductor parameter perturbation is rewritten to obtain the current discrete transfer function under the condition of constant expected voltage before and after parameter perturbation, and stability analysis is performed on the Z-transformed transfer function. Afterwards, in order to mitigate the instability issue caused by perturbations of the inductance parameters resulting from the implementation of a current prediction algorithm, it is proposed to enhance the voltage prediction equation within the traditional algorithm through utilization of weighted coefficients for optimization of direct-quadrature axis current equations. This optimization is intended to establish a correlation between the pole distribution of the discrete transfer function of the current before and after parameter perturbation and the weight coefficients. Finally, the current oscillations are reduced by varying the weighting coefficients to increase the range of the inductance parameters, which improves the stability of the motor fault tolerant control system, providing the motor a wider range of stable operation.

The simulation and experimental results show that the current oscillation range of the current prediction algorithm model with the addition of weighting coefficients during motor out-of-phase operation is significantly improved compared with the conventional current prediction algorithm. In addition, the sensitivity of the system stability to the inductor parameters is significantly reduced.
